# *Leishmania* infection upregulates and engages host macrophage Argonaute 1, and system-wide proteomics reveals Argonaute 1-dependent host response

**DOI:** 10.3389/fimmu.2023.1287539

**Published:** 2023-11-30

**Authors:** Atieh Moradimotlagh, Stella Chen, Sara Koohbor, Kyung-Mee Moon, Leonard J. Foster, Neil Reiner, Devki Nandan

**Affiliations:** ^1^ Division of Infectious Diseases, Department of Medicine, University of British Columbia, Vancouver, BC, Canada; ^2^ Department of Biochemistry and Molecular Biology, University of British Columbia, Vancouver, BC, Canada

**Keywords:** *Leishmania*, host-pathogen interactions, parasitic infection, RNAi, proteomics

## Abstract

*Leishmania donovani*, an intracellular protozoan parasite, is the causative agent of visceral leishmaniasis, the most severe form of leishmaniasis in humans. It is becoming increasingly clear that several intracellular pathogens target host cell RNA interference (RNAi) pathways to promote their survival. Complexes of Argonaute proteins with small RNAs are core components of the RNAi. In this study, we investigated the potential role of host macrophage Argonautes in *Leishmania* pathogenesis. Using Western blot analysis of *Leishmania donovani*-infected macrophages, we show here that *Leishmania* infection selectively increased the abundance of host Argonaute 1 (Ago1). This increased abundance of Ago1 in infected cells also resulted in higher levels of Ago1 in active Ago-complexes, suggesting the preferred use of Ago1 in RNAi in *Leishmania*-infected cells. This analysis used a short trinucleotide repeat containing 6 (TNRC6)/glycine-tryptophan repeat protein (GW182) protein-derived peptide fused to Glutathione S-transferase as an affinity matrix to capture mature Ago-small RNAs complexes from the cytosol of non-infected and *Leishmania*-infected cells. Furthermore, Ago1 silencing significantly reduced intracellular survival of *Leishmania*, demonstrating that Ago1 is essential for *Leishmania* pathogenesis. To investigate the role of host Ago1 in *Leishmania* pathogenesis, a quantitative whole proteome approach was employed, which showed that expression of several previously reported *Leishmania* pathogenesis-related proteins was dependent on the level of macrophage Ago1. Together, these findings identify Ago1 as the preferred Argonaute of RNAi machinery in infected cells and a novel and essential virulence factor by proxy that promotes *Leishmania* survival.

## 1 Introduction

Infection with intracellular pathogens belonging to the genus *Leishmania* results in a broad spectrum of clinical manifestations, mainly divided into three distinct clinical syndromes: visceral, cutaneous, and mucocutaneous leishmaniasis (1). *Leishmania donovani*, the focus of this study, causes visceral leishmaniasis, which can be life-threatening if left untreated (2). *Leishmania* has a digenetic life cycle, alternating between the non-motile amastigote form in the vertebrate host and the motile promastigote form in the invertebrate vector, the phlebotomine sandfly (3). The infection is initiated when an infected female sandfly injects metacyclic promastigotes into a mammalian host during a regular blood meal. Parasites released are rapidly taken up by phagocytic cells, including macrophages and neutrophils (4). The macrophages are the final resident cells which support *Leishmania* growth and proliferation, establishing chronic infection in humans. After internalization, promastigotes settle in phagolysosomes of macrophages, where they differentiate into amastigote forms, which divide many times and ultimately rupture macrophages and infect other surrounding macrophages (1).

Leishmaniasis is responsible for severe morbidity and mortality in parts of the tropics, subtropics and southern Europe. Leishmaniasis is classified as a neglected tropical disease. Unfortunately, leishmaniasis is rising due to increased global travel and a lack of effective therapeutic and prophylactic methods. Available drugs are expensive, have severe toxicity, and are circumvented by emerging parasite resistance (5). Although tremendous effort has been made to develop *Leishmania* vaccines, only some have been commercially approved for canine leishmaniasis (6), while none have yet been licensed for human leishmaniasis (5). Expanding the knowledge on the biological interaction between the parasite and the host is crucial for developing a comprehensive strategy to fight the disease and improve leishmaniasis treatment. The pathogenesis of *Leishmania* involves the alteration of the macrophage phenotype, where *Leishmania* causes deactivation of the host by interfering with host-cell gene expression and signal transduction (7). One potential mechanism of interest of *Leishmania* pathogenesis is targeting host small non-coding RNAs (sncRNAs), which are involved in post-transcriptional gene expression regulation, known as RNA interference (RNAi).

It is becoming increasingly clear that non-coding RNAs (ncRNAs), which do not encode proteins but account for the majority of the human genome, play important roles in many biological processes and diseases (8–11). Among them are small ncRNAs, such as microRNAs (miRNAs), Piwi-interacting RNAs, and tRNA-derived RNA fragments (tRFs), which have been shown to play a critical role in cancer, stress response, metabolic abnormalities and viral infections (12–16). Out of all species of ncRNAs, sncRNAs represent the core of the regulatory mechanisms of gene expression in eukaryotic cells. The mechanisms of action of most sncRNAs require a group of effector proteins as part of an RNA-induced silencing complex (RISC). These effector proteins are necessary for the stabilization, transport and regulatory activity of ncRNAs on their target RNA transcripts. RNA-binding proteins Argonautes (Agos) are members of the Argonaute family that bind sncRNAs that act as guide RNA and direct them to specific RNA transcripts for silencing. In fact, these highly conserved proteins are central components of the RISC in RNAi (17, 18). There are eight Ago family members in humans consisting of four ubiquitously expressed Ago1-4 of the Ago clade and four of the PIWI clade (PIWIL1-4), which are mainly restricted to the germline (19). Deep sequencing of human Ago-associated sncRNAs revealed Ago association with RNA fragments of diverse origin, such as miRNAs, small nucleolar RNAs, vault RNAs, and tRFs, to carry out their gene silencing function (20).

Ago proteins are transiently held in open conformation by heat shock proteins (HSPs) to allow loading of sncRNA, resulting in the closed conformation Ago-sncRNA complex, also known as the mature RISC. The mature RISC further interacts with the scaffolding protein trinucleotide repeat containing 6 (TNRC6)/glycine-tryptophan repeat protein (GW182), which preferentially binds to mature RISCs over guide-free Ago proteins (21) and facilitates downstream silencing processes, possibly by recruiting other RNA binding proteins (RBPs) (22). Finally, the RISC interacts with and represses expression of messenger RNA (mRNA) targets whose sequences are partially or fully complementary to those of the sncRNAs in the RISC.

Small ncRNAs (sncRNAs), such as microRNAs (miRNAs) and tRNA-derived RNA fragments (tRFs), have been clearly demonstrated to play an important role in microbial infections (12–14, 23). Our recent study showed that *Leishmania* infection causes genome-wide attenuation of macrophage miRNAs by decreasing miRNAs gene transcription. miRNA downregulation was linked to the upregulation of macrophage transcription factor c-Myc. c-Myc silencing reversed the *Leishmania*-mediated miRNA suppression and also reduced the intracellular survival of *Leishmania* (24). We have recently discovered that sncRNAs like tRFs and ribosomal RNA-derived RNA fragments (rRFs) are highly enriched in exosomes secreted by *L. donovani* and *L. braziliensis* (25), and these secretory exosomes have been shown to modulate innate and adaptive immune responses through predominantly immunosuppressive effects, such as promoting IL-10 production and inhibiting TNF-alpha in human monocytes and monocyte-derived dendritic cells (26). Overall, the emerging role of sncRNAs (such as miRNAs) from both host and pathogen (such as tRFs) during *Leishmania* infection represents a novel virulence paradigm that invites further examination. Thus, it was of interest to investigate the possibility that *Leishmania* targets host macrophage Ago(s) to regulate host gene expression to facilitate its survival.

In this study, we show that *L. donovani* selectively increases the abundance of host macrophage Ago1. Strikingly, the enhanced level of Ago1 in infected cells correlated with an increased level of Ago1 containing-complexes isolated using a recently published biochemical isolation of Argonaute protein complexes by “Ago proteins Affinity Purification by Peptide” (Ago-APP). Deliberate silencing of host Ago1 significantly attenuated intracellular survival of *Leishmania* in human macrophages, demonstrating that Ago1 is essential for *Leishmania* pathogenesis. SILAC (stable isotope labeling using amino acids in cell culture)-based quantitative proteomic analysis revealed that the expression of several previously reported *Leishmania* pathogenesis-related proteins was dependent on the level of macrophage Ago1, suggesting the role of Ago1 in *Leishmania* pathogenesis. Together, these findings identify Ago1 as the preferred Argonaute of RNAi machinery in infected cells and a novel and essential virulence factor by proxy that promotes *Leishmania* survival.

## 2 Materials and methods

### 2.1 THP-1 cell culture and differentiation

THP-1 cells (human leukemia monocytic cells) were obtained from ATCC (TIB-202TM), and maintained at 37°C, 5% CO_2_ in RPM1 1640 media (Gibco) supplemented with 10% heat-inactivated fetal bovine serum (Gibco), 10 mM HEPES (MilliporeSigma), 100 U/ml penicillin/streptomycin (MilliporeSigma) and 2 mM L-glutamine (MilliporeSigma). For differentiation, THP-1 cells were treated with 10 ng/ml Phorbol 12-myristate 13-acetate (PMA) overnight (16–18 h). Adhered differentiated THP-1 (dTHP-1) cells were washed with Hanks’ balanced salt solution (HBSS) (MilliporeSigma) and given fresh media without PMA. The cells were rested for 24 h prior to further treatment.

### 2.2 Parasite culture and *in vitro* infection

*Leishmania donovani* Sudan strain 2S was provided by Dr. Kwang Poo Chang (Rockefeller University, NY, USA), and continuously cultured in the lab. Virulence was maintained by regular passages via Syrian Golden hamster from which fresh amastigotes were purified and transformed *in vitro* into promastigotes (24). Promastigotes were cultured in M199 medium (MilliporeSigma) supplemented with 10% heat-inactivated fetal bovine serum, 10 mM HEPES, 10 µg/ml folic acid (MilliporeSigma), 3 µg/ml hemin (MilliporeSigma), 2 mM L-Glutamine, 100 U/ml penicillin/streptomycin and 100 mM adenosine (MilliporeSigma) at 26°C. The parasites were subcultured every three days for a maximum of 25-30 passages. For *Leishmania* infections, stationary phase promastigotes were used at an MOI of 20:1 unless otherwise stated.

### 2.3 Determination of infection rate

To determine the infection rate, *Leishmania*-infected cells were washed with warm PBS, then fixed with 2% paraformaldehyde in PBS for 20 min on ice, and mounted on glass slides using VECTASHIELD Antifade Mounting Medium with DAPI, which stains macrophage and parasite nuclei. Cells were viewed under fluorescence using the Axioplan II (Carl Zeiss Inc.) microscope. For each treatment, at least 10 images were taken at 40x magnification using the AxioCam MRm Camera and the AxioVision software Version 4.8.2 (Carl Zeiss Inc.). At least 100 cells were counted for each condition to determine the average number of parasites per macrophage and the percentage of macrophages infected.

### 2.4 Parasite rescue and transformation assay

PMA differentiated THP-1 cells (dTHP-1) were infected with stationary-phase *L. donovani* promastigotes at an MOI of 20:1 for 24 h. After the duration of infection, cells were thoroughly washed with HBSS to remove non-internalized *Leishmania*. Controlled lysing of infected cells was performed using 0.01% SDS as previously described (24, 27) to release intracellular amastigotes. The rescued *Leishmania* amastigotes were transformed to promastigotes in M199 media by incubating at 26°C for 72 h. Their growth was evaluated after 72 h of incubation by counting transformed promastigotes stained with trypan blue solution (0.4% w/v in PBS) on a hemocytometer.

### 2.5 Western blotting

dTHP-1 cells were washed with HBSS and lysed in lysis buffer (20 mM Tris-HCl, pH 7.5, 150 mM NaCl, 1% Triton X-100, 5 mM NaF, 1 mM Na_3_VO_4,_ 1 mM EDTA), supplemented with 1 mM PMSF, 5 µg/ml aprotinin and 5 µg/ml leupeptin. Whole cell lysates were clarified by centrifugation and proteins in supernatant were separated by SDS-PAGE and transferred to nitrocellulose membranes (Bio-Rad) using a semi-dry transfer apparatus. Transferred proteins were probed with the appropriate antibodies, according to the manufacturer’s instructions: Anti-Ago1 (Cell Signaling 5053), anti-Ago2 (Cell Signaling 2897), anti-GST (Santa Cruz sc-138), anti-GAPDH (Applied Biological Materials (Abm) G041), anti-Lamin A/C (Cell Signaling 2032), and anti-Actin (Santa Cruz SC-47778). Reactive protein signals were captured on Blue X-ray film (Carestream) using SuperSignal West Pico Plus or Femto Chemiluminescence Substrate (Thermo). Densitometric analysis was performed in ImageJ, and results were normalized to Actin.

### 2.6 THP-1 cell culture in SILAC media

For SILAC-based labeling, RPMI 1640 media without L-glutamine, L-arginine and L-lysine (Caisson labs) was supplemented with 10% heat-inactivated dialyzed fetal bovine serum (Gibco), 2 mM L-glutamine, 10 mM HEPES and 100 U/ml penicillin/streptomycin. The RPMI medium was then split into three: the “light” RPMI was supplemented with normal isotopic abundance arginine (21 mg/l) and lysine (36.5 mg/l), while the “medium” RPMI was supplemented with ^13^C_6_-arginine (20.25 mg/l) and ^2^H_4_-lysine (37.5 mg/l), and “heavy” RPMI was supplemented with ^13^C_6_^15^N_4_-arginine (22.25 mg/l) and ^13^C_6_^15^N_2_-lysine (38.5 mg/l).

THP-1 cells were spun down, one third of which were resuspended in the respective SILAC media, incubated at 37°C and 5% CO_2_, and passaged for a minimum of five times prior to use. Cells were differentiated using 10 ng/ml of PMA for 16-18 h and then washed with HBSS and rested for 24 h in fresh respective SILAC media prior to infection.

### 2.7 siRNA-mediated knockdown of Ago1 in THP-1 cells

Three siRNAs targeting human Ago1 (SR308857), as well as non-specific control (scrambled) siRNA (SR30004), were purchased from OriGene. Cells were transfected for 52 h with non-specific or Ago1 siRNA (50 pmol/well) using HiPerFect transfection reagent (Qiagen) according to the manufacturer’s instructions. We have previously used HiPerFect transfection reagent (Qiagen) to silence the expression of various proteins in THP-1 cells (28) and in human monocyte-derived macrophages (24) using siRNAs from OriGene, with transfection efficiency between 70-80%, as measured by fluorescent-labeled transfection control siRNA (OriGene). After the duration of transfection, THP-1 cells were differentiated with PMA for further use.

### 2.8 Expression and purification of recombinant proteins

For the expression and purification of required recombinant proteins we used a previously published protocol (29). Briefly, GST(glutathione S-transferase) and GST-T6B plasmid constructs were transformed in DH5 alpha *E. coli* and then expressed in BL21. Subsequently, 2 L isopropyl β-D-1-thio- galactopyranoside–induced culture (OD_600 _= 0.6) was grown overnight at 18°C. The cells were harvested by centrifugation at 4,400 × g, 30 min at 4°C and the resulting pellet was then resuspended in resuspension buffer (PBS containing 1 mM PMSF and 1 mM DTT) supplemented with 1 mg/ml lysozyme. After sonication for 4 × 30 sec, the lysate was cleared by centrifugation at 40,000 × g, 40 min at 4°C. Then the supernatant was incubated with 3 ml washed Glutathione-Agarose beads (MilliporeSigma) at 4°C for 3 h. Thereafter, the GST/GST-T6B-coupled beads were loaded onto a column and washed with 10 column volumes (cv) of resuspension buffer. GST/GST-T6B was eluted with 4 cv of elution buffer (PBS supplemented with 20 mM Tris-HCl, pH 8.0, and 10 mM reduced glutathione), pooled and then concentrated with Amicon Ultra-15 centrifugal filter units. The concentrated fractions were loaded onto a desalting chromatography column (Zeba™ Spin Desalting Columns) that was equilibrated in PBS with 5% (v/v) glycerol. Desalted fractions were adjusted to ∼2 mg/ml proteins, aliquoted and stored at −80°C. The purity of recombinant proteins was assessed by staining with Coomassie brilliant blue.

### 2.9 Preparation of cytosolic fraction

For the preparation of the cytosolic fraction, we followed a published protocol (30). Briefly, dTHP-1 cells were extensively washed and lysed in hypotonic lysis buffer (10 mM Tris-HCl, pH 7.5, 10 mM NaCl, 3 mM MgCl_2_, 0.3% NP-40, 1 mM NaF, 1 mM Na_3_VO_4_) supplemented with 5 µg/ml aprotinin, 5 µg/ml leupeptin, and 1 mM PMSF, and passed ten times through a 22-gauge needle on ice to disrupt cells. Then disrupted cells were centrifuged at 2,300 × g for 20 min at 4°C. The supernatant was kept as the cytoplasmic fraction. The purity of cytoplasmic fractions was assessed using antibodies against specific markers for the cytoplasm (GAPDH) and nuclear fraction (Lamin A/C) in a Western blot assay. These cytoplasmic fractions were used for the purification of Agos and associated proteins using GST-T6B affinity beads.

### 2.10 GST-T6B affinity chromatography

To prepare the affinity beads, 50 µl of Glutathione-Agarose beads were incubated with approximately 100 µg of GST/GST-T6B recombinant proteins for 3 h at 4°C with end-over-end rotation. Non-bound peptides were removed by washing the beads three times with excess of ice-cold PBS. In order to remove non-specific proteins which might have interacted with GST alone or Glutathione-Agarose beads, the cell lysates of interest prepared in NET buffer (50 mM Tris, pH 7.5, 150 mM NaCl, 5 mM EDTA, 0.5% NP-40, 10% glycerol, 1 mM NaF; supplemented with 0.5 mM DTT and 1 mM PMSF) were pre-incubated with 50 µl of Glutathione-Agarose beads loaded with 100 µg GST for 1 h at 4°C with end-over-end rotation. Non-bound materials were then incubated with the GST-T6B affinity beads for 3 h, at 4°C to capture interacting proteins. Beads were spun down, washed three times with NET buffer to remove non-bound material, and once with PreScission cleavage buffer (50 mM Tris, pH 7.5, 150 mM NaCl, 1 mM EDTA, 1 mM DTT). The affinity beads were eluted by PreScission enzyme (MilliporeSigma) cleavage at 4°C or by addition of 2x Laemmli sample loading buffer and incubation at 95°C for 7 min.

### 2.11 Liquid chromatography-tandem mass spectrometry and protein identification

For mass spectroscopy analysis of siRNA-mediated Ago1 downregulated macrophages, equal amounts of proteins from each treatment were mixed, reduced, alkylated (31) and run on 10% SDS-PAGE. Proteins in the entire lane were digested in gel with MS-grade trypsin (Promega) (32) and resulting peptides were cleaned on C-18 STop And Go Extraction (STAGE) tips (33) using 40% (v/v) acetonitrile in 0.1% (v/v) formic acid as the elution buffer. Peptides were analysed on NanoDrop One (Thermo Scientific - A205, scopes) to load approximately 200 ng of peptides on Orbitrap Exploris 480 coupled to easy nLC 1200 (Thermo Scientific) with ionopticks’ Aurora series 25 cm x 75 µm C18 1.6 µm analytical column heated to 40°C. Peptides were separated on a 62 minute gradient with a LC flow rate of 250 nl/min. Buffer A consisted of 2% (v/v) acetonitrile and 0.1% (v/v) formic acid; buffer B consisted of 80% (v/v) acetonitrile and 0.1% (v/v) formic acid. Spray voltage was set to 1,900 V and ion transfer tube temperature to 290°C. FAIMS was not enabled with data acquisition. Data dependent mode was set for 20 scans and the orbitrap resolution was set to 120,000 for full MS and 15,000 for fragment MS, with AGC target set to 100% at 20 ms for full MS and 50% for fragment MS. Intensity threshold was set at 8,000, isolation window at 2 m/z, with normalized collision energy at 28%. Dynamic exclusion was enabled to exclude after 1 time for 45 s.

Acquired data were searched on MaxQuant version 2.1.0.0 (34) against Uniprot’s human sequences (UP000005640), *Leishmania donovani* BPK282A1, and common contaminant sequences. SILAC labels of medium arginine (^13^C_6_), medium lysine (D_4_), heavy arginine (^13^C_6_, ^15^N_4_), and heavy lysine (^13^C_6_, ^15^N_2_) were set for quantitation enabling iBAQ, requantify, and match-between-runs options. The data were filtered for 1% false discovery at protein, peptide and PSM levels.

Proteomics data have been deposited to the ProteomeXchange Consortium via the PRIDE partner repository with the dataset identifier PXD037042. The reviewer account for the PRIDE data has the username reviewer_pxd037042@ebi.ac.uk and password o1Lv0bxR.

### 2.12 Bioinformatic analysis of LC-MS/MS data

All downstream analysis was done using R software (http://www.r-project.org). Of the total protein groups (hereafter referred to simply as ‘proteins’) detected by LC-MS/MS, we removed those that were commonly occurring contaminants, identified by peptides found to be part of a protein derived from the reversed part of the decoy database, or non-human, yielding a filtered high-confidence dataset of human proteins. Proteins were further filtered to only keep those with a minimum of two unique peptides per protein identification and which were identified in at least two of the three replicates. For all analyses, log_2_ transformed normalized ratios (fold changes) were used.

The one-sample T-test was performed to statistically compare log_2_ transformed normalized ratios to the hypothetical value of 0 (representing no change). A p-value of less than 0.05 was considered statistically significant. No cut-off was set as the minimum fold change for a protein to be considered modulated. Instead, where applicable, the significance of each protein modulation was ranked by distance from the origin measured along axes (Manhattan distance), when represented in a volcano plot of -log_10_(p-value) vs. log_2_(fold change). The higher the Manhattan score, the more significant the modulation of the protein was considered. For proteins that were both *Leishmania*-modulated and Ago1-dependent, the sum of their Manhattan scores was used to rank their combined significance.

We compared our dataset with the Pathways Commons collection of databases (https://www.pathwaycommons.org/), which collects biological pathway and interaction data from various partner databases—including, but not limited to, BioGRID, SwissProt, NCI Pathway Interaction Database: Pathway, and Innate DB.

For Gene Ontology (GO) annotation, Uniprot Princeton GO Mapper (https://go.princeton.edu/cgi-bin/GOTermMapper) was used with the following parameters: Organism (Annotation) set to “Homo sapiens (GOA @EBI + Ensembl)” and Ontology set to “Generic slim”. The proteins were annotated for each of three biological aspects: Biological process (“process”), Molecular function (“function”), and Cellular component (“component”). We omitted GO terms with 3 or fewer protein members or that were very broad and encompassed many subcategories (“Organelle”, with 68/71 proteins). Conversely, we combined similar highly specific GO terms (“Catalytic activity acting on a protein”, “Catalytic activity acting on RNA”, “activity acting on DNA”) into their corresponding umbrella term (“Catalytic activity”). Finally, for sets of two nearly identical GO terms, we retained only one (“Extracellular space” but not “Extracellular region”, and “Extracellular matrix” rather than “External encapsulating structure”).

## 3 Results

### 3.1 The host Ago1 is upregulated during *Leishmania* infection and downregulation of Ago1 attenuates *Leishmania* survival

**Figure 1:**
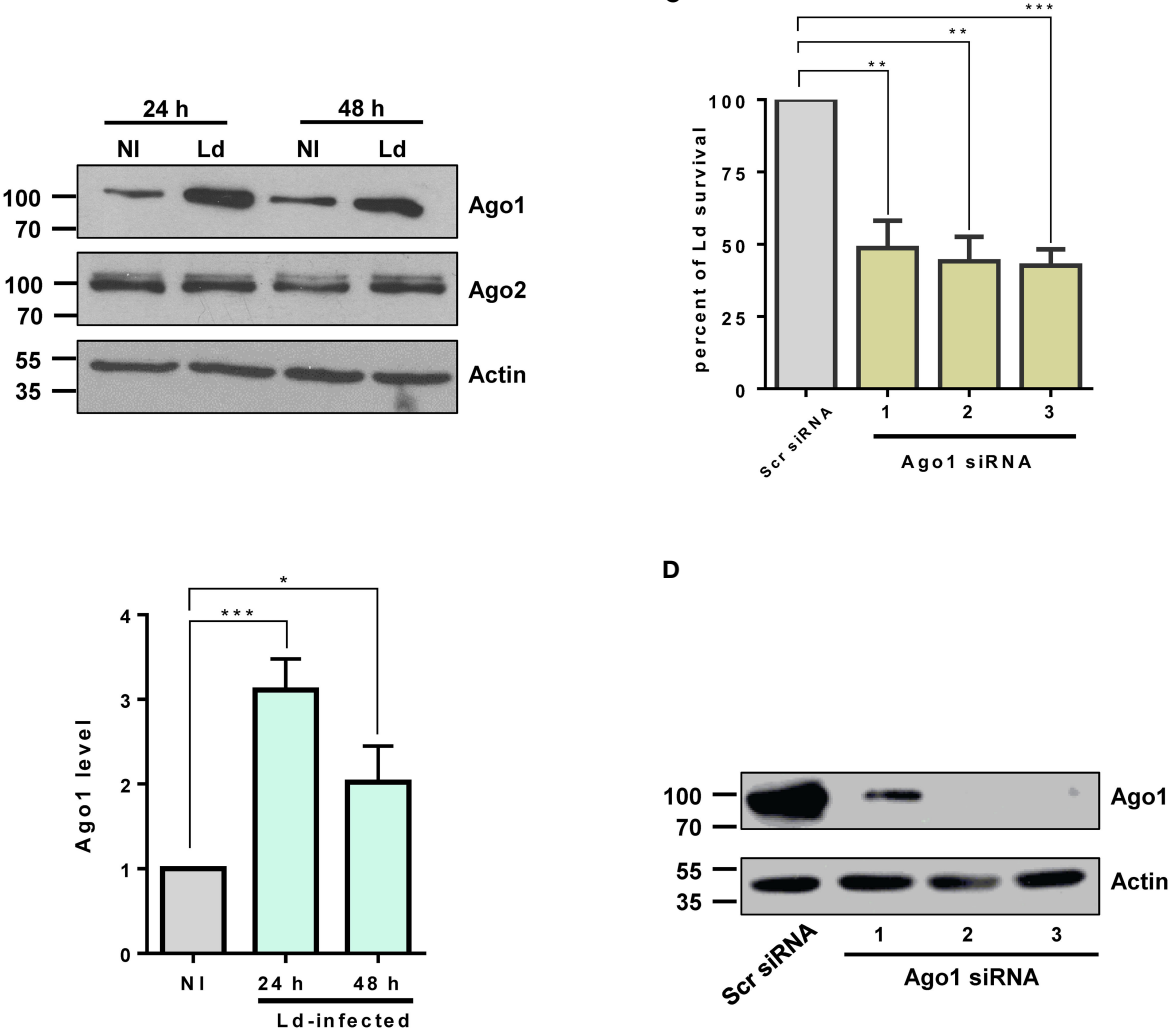
Ago1 is upregulated in infected macrophages. **(A)** dTHP-1 cells were infected with *L. donovani* (Ld) or not for 24 h and 48 h, whole cell lyates were from non-infected (NI) and Ld-infected macrophages were analysed by Western blotting with antibodies specific for Ago1, Ago2 and Actin (loading control). **(B)** Densitometric analysis was used to measure Ago1 and Ago2, normalized to Actin. The histogram shown is the mean ± S.D. of the densitometric analyses of three independent experiments. **(C)** Ago1 knockdown attenuates *Leishmania* survival. THP-1 cells were treated with control scrambled siRNAs or three different Ago1-specific siRNAs for 52 h Control and Ago1-knockdown cells were differentiated and subsequently incubated with *Leishmania* for 24 h At the end of the experiment, a parasite rescue assay as described in “Materials and methods” was performed. Shown is the mean ± S.D. of the densitometric analyses of three independent experiments (*p-value < 0.05, **p-value < 0.01, ***p-value < 0.001). **(D)** In parallel, the cells treated with siRNAs and differentiated for parasite rescue assay were analyzed by Western blotting for Ago1 and Actin levels.

Argonautes 1 and 2 are the most abundant in mammalian cells and have been extensively studied in various biological contexts such as regulation of the biogenesis of small RNA, mRNA translation, small RNA-guided host defense against exogenous nucleic acids, genome editing and even disease progression (17). Thus, we investigated the modulation of host macrophage Ago1 and Ago2 in response to *L. donovani* infection in PMA-differentiated THP-1 cells (dTHP-1). These cells have been extensively used as a model system to study human leishmaniasis (35–37). To examine Ago1 and Ago2 levels, dTHP-1 cells were infected with stationary-phase *L. donovani* promastigotes at MOI 20:1 for 24 h and 48 h. Ago1 and Ago2 levels were monitored by Western blot assay using their respective antibodies. As shown in **Figures 1A, B**, the expression level of Ago1 was selectively and strongly upregulated, while the Ago2 level remained unaltered in infected cells, both at 24 and 48 h post-infection. We checked the specificity of commercial Ago1 antibody from Cell signaling using whole cell lysate of dTHP-1 cells (positive control) and whole cell lysate of *Leishmania* promastigotes in a Western blot assay. Anti-Ago1 showed no detectable reactivity in the whole cell lysate of *Leishmania* promastigotes (data not shown), thus rejecting any potential contribution of *Leishmania* Ago1 in the observed upregulation of host Ago1 in infected cells. Furthermore, to rule out the possibility that *Leishmania*-induced Ago1 is due to the general phagocytosis of external particles, we incubated dTHP-1 cells with latex beads at different MOIs (5:1, 10:1 and 20:1) for 24 h. Western blot analysis of the bead-treated cells for Ago1 level revealed no significant induction of Ago1 compared to non-treated control cells (**Figure S1**). This strongly suggests that the increase in Ago1 abundance following *Leishmania* infection is not mediated by general phagocytotic processes.

Given that Ago1 binds a variety of sncRNAs, the central component of RISC in the RNA silencing pathway, it seemed likely that Ago1 upregulation would have pleiotropic and important effects on the macrophage phenotype. In addition, it seemed probable that this upregulation of Ago1 itself, independent of any impact on the RNAi pathway, would have consequences for macrophage biology. Thus, to address the biological relevance of *Leishmania*-mediated selective increase in host Ago1 level, we investigated the possibility that Ago1 may confer a pathogen survival advantage. We therefore assessed *Leishmania* intracellular survival in host macrophages treated with Ago1 siRNAs. To this end, we downregulated host Ago1 using three unique Ago1-targeting siRNAs. A non-specific scrambled siRNA was used as a negative control. Ago1-knockdown cells and control cells were infected with *L. donovani* for 24 h. At the end of the experiment, the infected cells were lysed for parasite rescue, as described in “Materials and methods.” The results presented in **Figure 1C** show that reduced Ago1 level correlated with decreased survival of *Leishmania.* In parallel, the downregulation of Ago1 was confirmed by immunoblotting using Ago1 antibody. The efficiency of siRNA-mediated Ago1 knockdown is shown in **Figure 1D**. All three unique siRNAs were able to downregulate Ago1. The transfection of siRNAs 2 and 3 resulted in nearly complete downregulation of Ago1. Together, these results show that host Ago1 is essential for the intracellular survival of *Leishmania*.

Results presented thus far show enhanced levels of Ago1 in infected cells. Deliberate downregulation of Ago1 resulted in decreased survival of *Leishmania* inside host cells. Given that Argonaute proteins participate in the RNA silencing pathway as the central component of RISC (17, 38), it was important to investigate the possibility that an enhanced level of Ago1 in infected cells also results in a higher level of Ago1-containing mature RISC complexes required for RNAi. Thus, we isolated Ago protein complexes from non-infected control and *Leishmania-*infected cells.

### 3.2 Preparation of GST-TNRC6 affinity beads and validation for its interaction with Argonautes

We used a recently published procedure, “Ago protein Affinity Purification by Peptides,” to isolate Ago-containing complexes from control and infected cells (29). This affinity column-based approach allows for the simultaneous isolation of all Argonautes and associated proteins from a variety of species and cell lines. This robust pull-down assay involves the (GST)-T6B peptide, a part of the TNRC6/GW182 protein family that efficiently interacts with all Ago proteins (29).

To prepare recombinant GST-T6B peptide and control affinity beads, purified GST-T6B proteins and GST alone were loaded respectively on Glutathione-Agarose beads as described in “Materials and methods.” The purity of concentrated and desalted recombinant proteins is shown in **Figure S2A**. Without antibodies against the T6B peptide, expected molecular mass and reactivity to anti-GST antibodies were used to correctly identify recombinant proteins (**Figure S2B**). To test that recombinant GST-T6B peptide is active and successfully isolates Ago proteins from macrophages, whole cell lysate was incubated with GST alone and GST-T6B affinity beads separately. Bound proteins were released by heating in the Laemmli sample buffer. The presence of Agos in the bound materials was investigated by immunoblotting using appropriate antibodies. As expected, Ago1 and Ago2 were detected in material eluted from GST-T6B beads but absent from material eluted from GST alone, thus validating the specificity and effectiveness of peptide affinity beads in the isolation of Ago proteins (**Figures S2C, D**).

### 3.3 Ago protein-containing complexes isolated from *Leishmania*-infected cells are enriched in Ago1

As described above, it was of interest to investigate whether this increase in Ago1 correlates with an increase in Ago1 being part of RISC, which is responsible for the RNAi process for gene regulation.

**Figure 2:**
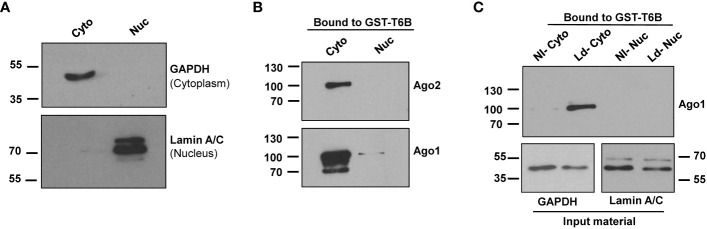
Analysis of cytoplasmic and nuclear fractions from non-infected and infected macrophages for the presence of Ago1. **(A)** dTHP-1 cells were subjected to cell fractionation for cytoplasmic and nuclear fractions as described in “Materials and methods” and the purity of fractions was assessed using indicated antibodies in a Western blot assay. **(B)** The cytoplasmic and nuclear extracts from dTHP-1 cells were incubated separately with GST-T6B affinity beads and bound proteins were subjected to Western blotting using indicated antibodies for the presence of Ago1 and Ago2. **(C)** Immunoblot comparing levels of Ago1 in bound materials eluted from GST-T6B affinity beads incubated with cytosolic or nuclear lysates of non-infected (NI) and *Leishmania*-infected (Ld) dTHP-1 cells. In parallel, aliquots from the cytoplasmic fraction from NI and *Leishmania-*infected cells were tested for GAPDH and Lamin A/C level. The data shown is one representative of three independent experiments.

It is well established that most Ago complexes are restricted to the cytoplasm (22, 39) to participate in canonical RNAi. Thus, we used cytoplasmic fractions to isolate Ago protein-containing complexes using GST-T6B affinity beads for further study. GAPDH was used as the cytoplasm marker, and Lamin A/C was used as the nuclear marker. The results presented in **Figure 2A** show cytoplasmic fraction nearly devoid of nuclear content. Bound complexes from GST alone and GST-T6B affinity beads were released by heating in Laemmli sample buffer. The presence of Agos in the bound materials was investigated by immunoblotting using appropriate antibodies. As expected and shown in **Figure 2B**, Ago1 and Ago2 are enriched in cytoplasm and nearly completely absent in the nuclear fraction. After confirmation of the enrichment of Agos in the cytoplasm, cytoplasmic and nuclear fractions from control and infected cells were separately incubated with GST-T6B affinity beads to isolate Ago-containing complexes. As shown in **Figure 2C**, the amount of Ago1 was strikingly enriched in complexes isolated from cytoplasmic fractions of *Leishmania-*infected cells compared to non-infected cells. This finding of Ago1 enrichment in RISC isolated from infected cells positively correlates with the abundance of Ago1 in *Leishmania*-infected cells. Taken together, results obtained suggest that *Leishmania* infection promotes Ago1-containing mature complexes, which are essential for RNAi.

The results thus far show that Ago complexes are enriched in Ago1-containing complexes, and host macrophage Ago1 is critical for optimal intracellular survival of *Leishmania*. Together these findings prompted us to further study the role of Ago1 in the pathogenesis of *Leishmania* infection. To this end, we used a comprehensive, unbiased SILAC-based quantitative proteomic analysis of *Leishmania*-infected cells in an Ago1-deficient condition.

### 3.4 SILAC-based quantitative proteomic analysis of *Leishmania*-infected macrophages in normal and Ago1-deficient condition

Our approach to comprehensively identify and characterize host macrophage proteins modulated by *Leishmania* and their dependence on Ago1 involves the gene silencing of host cell Ago1 by siRNAs unique to human Ago1, followed by SILAC-based quantitative LC-MS/MS analysis (outlined in **Figure S3A**). For SILAC-based quantitative analysis, THP-1 cells were cultured in corresponding SILAC media as shown in **Figure S3A**. The “light” (L) cell population was transfected with control siRNA (non-infected). The “medium” (M) cell population was transfected with control siRNA and infected with *L. donovani*. The “heavy” (H) cell population was transfected with Ago1-specific siRNAs and infected with *L. donovani*. Due to the restricted availability of SILAC isotopes, we elected to pool two unique siRNAs (#2 and #3, as shown above in **Figure 1D**). As expected and shown in **Figure S3B**, pooling of unique siRNAs was effective in downregulating host Ago1, and the use of control siRNA did not prevent *Leishmania*-mediated upregulation of Ago1. After 24 h incubation with *Leishmania*, cells were lysed, and equal amounts of proteins from “light,” “medium” and “heavy” populations were mixed in a 1:1:1 ratio. Proteins were subsequently identified and quantified by liquid chromatography-tandem mass spectrometry (LC-MS/MS) as described in “Materials and methods”.

### 3.5 Global proteomic quantitation-based identification of *Leishmania*-modulated host proteins in infected macrophages

**Figure 3:**
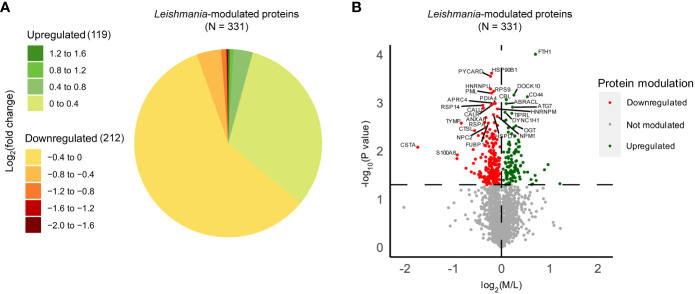
Quantitative proteomic analysis of non-infected and *Leishmania*-infected macrophages. THP-1 cells were cultured in SILAC media as described in “Materials and methods”. Labeled THP-1 cells were treated with an equal mix of two Ago1 siRNAs or with control scrambled siRNA. Control and Ago1-knockdown cells were differentiated and incubated with *Leishmania* for 24 h. Subsequently, control and infected cells were washed extensively and mass spectrometry was performed on a mix of three lysates allowing for a quantitative comparison between non-infected, infected cells and infected in Ago1 knockdown condition (three independent experiments). Shown is the comparison between non-infected (L) and *Leishmania*-infected (M) cells. **(A)** Pie chart of the log_2_(fold change) distribution of *Leishmania*-modulated proteins. M/L normalized ratios were log_2_ transformed and averaged for at least 2 replicates. P-values < 0.05 from one-sample T-tests were considered significant. Proteins with a negative log_2_(fold change) were downregulated in *Leishmania*-infected cells compared to non-infected cells, whereas proteins with a positive log_2_(fold change) were upregulated in *Leishmania*-infected cells compared to non-infected cells. **(B)** Volcano plot comparing protein expression levels in *Leishmania*-infected cells with those in non-infected cells, both treated with control siRNA. Proteins significantly upregulated by *Leishmania* infection are shown as green dots, and those that were downregulated, as red dots. The horizontal dashed line marks the p-value cut off of 0.05 or -log_10_(p-value) = 1.301. The vertical dashed line represents log_2_(M/L) = 0. L, Light (Scrambled siRNA-treated, Non-infected); M, Medium (Scrambled siRNA-treated, Ld-infected).

In order to identify *Leishmania*-modulated proteins which are dependent on Ago1 and which may have relevance to *Leishmania* pathogenesis, we first identified host macrophage proteins that were differentially expressed after *Leishmania* infection. As shown in **Figure S4A**, we filtered the initial 3379 proteins identified through LC-MS/MS to retain only high-confidence human proteins, resulting in 2151 proteins. Of these, we considered only those with ratios detected in at least two out of three replicates. We performed one-sample T-tests comparing the log_2_(M/L) ratios to zero, which represents no change in response to *Leishmania* infection. Of the total 1778 proteins identified, 331 were significantly modulated by *Leishmania* (**Table S1**). 212 of the differentially expressed proteins were downregulated, while 119 proteins were upregulated in response to *Leishmania* infection (**Figure 3A**). For the ease of readers, *Leishmania*-modulated proteins were also represented in a volcano plot, where the -log_10_(p-value) was plotted against the log_2_(M/L) ratio (**Figure 3B**).

For in-depth analysis in terms of their biological relevance, we considered the top 30 host proteins ranked by Manhattan distance (as described in “Materials and methods”) that were significantly modulated by *Leishmania* (**Table 1**). Interestingly, 14/30 have been shown to be implicated in *Leishmania* infection-related studies, such as Ferritin heavy chain (FTH1), Cystatin-A, Apoptosis-associated speck-like protein containing a CARD (PYCARD), CD44 antigen (CD44) and Heterogeneous nuclear ribonucleoprotein U (HNRNPU). Together, our proteomic analysis of differentially expressed proteins in *Leishmania*-infected host cells exhibits significant overlap with those that have been previously shown to be implicated in *Leishmania* infection, thus providing added confidence to our quantitative proteomic analysis.

**Table 1:** Top 30 *L. donovani* infection-modulated proteins.

Protein names	Gene names	Uniprot ID	Average log_2_(M/L)	P-value log_2_(M/L)	Manhattan distance	Known roles related to *Leishmania*
**Ferritin heavy chain;Ferritin heavy chain, N-terminally processed;Ferritin**	FTH1	P02794	0.703	1E-04	4.714	*L. amazonensis* infection causes iron accumulation and ferritin upregulation in macrophages (40)
**Endoplasmin**	HSP90B1	P14625	-0.205	2E-04	3.821	N/A
**Cystatin-A;Cystatin-A, N-terminally processed**	CSTA	P01040	-1.737	0.008	3.818	Cystatin cured experimental visceral leishmaniasis by switching the differentiation of Th2 cells to Th1 type, as well as upregulating NO (41); cystatin and IFN-γ inhibited the growth of amastigotes in macrophages (41)
**Apoptosis-associated speck-like protein containing a CARD**	PYCARD	Q9ULZ3	-0.228	3E-04	3.780	*L. major* infection downregulates PYCARD (42); PYCARD transcript upregulation observed in *L. infantum*-infected dog glomeruli and tubules (43); PYCARD downregulated in *L. amazonensis*-infected macrophages, but upregulated in *L. major*-infected cells (44)
**CD44 antigen**	CD44	P16070	0.534	7E-04	3.663	*Leishmania*-encoded migration inhibitory factor (LmMIF) upregulated both CD74 and its associated coreceptor, CD44 (45)
**Heterogeneous nuclear ribonucleoprotein U**	HNRNPU	Q00839	-0.228	5E-04	3.519	HNRNPU was upregulated at the mRNA level by *L. donovani* amastigotes, while its levels were unchanged by *L. donovani* promastigotes (46); An increased abundance of proteins involved in RNA splicing (heterogeneous nuclear ribonucleoproteins [hnRNPs]) was observed at 24 h post-infection with *L. donovani* (36)
**Dedicator of cytokinesis protein 10**	DOCK10	Q96BY6	0.258	7E-04	3.423	N/A
**Thymidine phosphorylase**	TYMP	P19971	-0.834	0.003	3.415	*L. major* (unlike *L. mexicana*) appears to metabolically incorporate 5F-uridine, probably through a uridine or thymidine phosphorylase (47); The major pathway of thymidine metabolism in *L. donovani* was cleavage of the deoxyriboside linkage to form thymine, probably via the action of a thymidine phosphorylase (48)
**Protein PML (TRIM19)**	PML	P29590	-0.205	6E-04	3.410	N/A
**40S ribosomal protein S9**	RPS9	P62263	-0.162	6E-04	3.405	Upregulated by *L. major* (49)
**40S ribosomal protein S14**	RPS14	P62263	-0.388	0.001	3.340	N/A
**Calumenin**	CALU	O43852	-0.378	0.001	3.270	N/A
**E3 ubiquitin-protein ligase CBL**	CBL	P22681	0.101	9E-04	3.168	N/A
**Calreticulin**	CALR	P27797	-0.212	0.001	3.164	N/A
**Actin-related protein 2/3 complex subunit 4**	ARPC4; ARPC4-TTLL3	P59998	-0.162	0.001	3.148	N/A
**Ubiquitin-like modifier-activating enzyme ATG7**	ATG7	O95352	0.231	0.001	3.143	N/A
**Protein disulfide-isomerase A4**	PDIA4	P13667	-0.129	0.001	3.119	Upregulated by *L. amazonensis* and *L. major* (50)
**Costars family protein ABRACL**	ABRACL	Q9P1F3	0.099	0.001	3.085	N/A
**Annexin A6;Annexin**	ANXA6	P08133	-0.324	0.002	3.018	N/A
**TIP41-like protein**	TIPRL	O75663	0.215	0.002	3.000	N/A
**Cathepsin L1;Cathepsin L1 heavy chain;Cathepsin L1 light chain**	CTSL	P07711	-0.559	0.004	2.985	Plays a role in the processing of soluble *Leishmania* antigen (SLA) in *L. major* (51); Treatment with cathepsin L inhibitor potentiates Th2-type immune response in *L. major*-infected BALB/c mice (52)
**Heterogeneous nuclear ribonucleoprotein M**	HNRNPM	P52272	-0.083	0.001	2.959	Host nucleoplasmic HNRNPM increased in abundance in a *L. major* GP63-dependent manner (53)
**40S ribosomal protein SA**	RPSA	P08865	-0.348	0.003	2.941	N/A
**60 kDa heat shock protein, mitochondrial**	HSPD1	P10809	-0.168	0.002	2.924	Upregulated in *L. amazonensis* infection (54)
**Cytoplasmic dynein 1 heavy chain 1**	DYNC1H1	Q14204	0.074	0.002	2.879	N/A
**Far upstream element-binding protein 1**	FUBP1	Q96AE4	-0.273	0.003	2.853	N/A
**Protein S100-A8;Protein S100-A8, N-terminally processed**	S100A8	P05109	-0.917	0.012	2.837	S100A8/S100A9 can act as a biomarker in experimental leishmaniasis for phagocyte activation linked to an effective Th1-response (55); S100A8 transcript was repressed upon *L. major* infection (56)
**Epididymal secretory protein E1**	NPC2	P61916	-0.334	0.003	2.825	*L. donovani* causes a downregulation of *npc2* and *npc1* genes involved in the uptake of extracellular cholesterol during establishment of infection (57)
**UDP-N-acetylglucosamine–peptide N-acetylglucosaminyltransferase 110 kDa subunit**	OGT	O15294	0.299	0.003	2.825	N/A
**Nucleophosmin**	NPM1	P06748	-0.089	0.002	2.809	Downregulated in the thymic interstitial fluid of mice infected with *L. infantum* (58)

### 3.6 Global identification of *Leishmania*-modulated Ago1-dependent host proteins in infected macrophages

**Figure 4:**
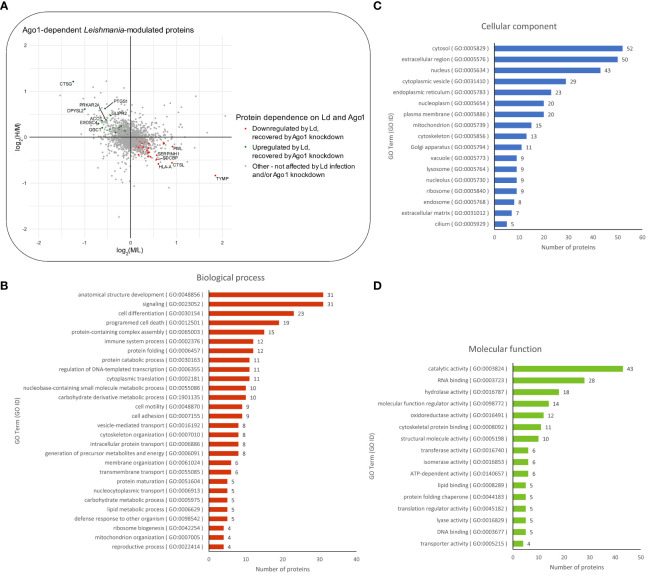
Proteomic analysis of Ago1-dependent *Leishmania*-modulated macrophage proteins. **(A)** Scatter plot of log_2_ transformed M/L ratios (*y*-axis) *vs*. log_2_ transformed H/M ratios (*x*-axis) of proteins. Proteins that were upregulated by *Leishmania* infection and recovered to any degree following Ago1 knockdown are shown in green. Proteins that were downregulated by *Leishmania*-infection and recovered to any degree following Ago1 knockdown are shown in red. The Ago1-dependent *Leishmania*-modulated proteins were also classified by GO terms in three categories: **(B)** biological process, **(C)** cellular component, and **(D)** molecular function. P-values < 0.05 from one-sample T-tests were considered significant for both log_2_ transformed M/L and H/M fold changes. L, Light (Scrambled siRNA-transfected, Non-infected); M, Medium (Scrambled siRNA-transfected, Ld-infected); H, Heavy (Ago1 siRNA-transfected, Ld-infected).

Next, we sought to determine which of the differentially expressed proteins in *Leishmania*-infected macrophages are dependent on host Ago1 by combining two sets of proteins as shown in **Figure S4B**: (i) proteins that are upregulated in response to *Leishmania* infection (log_2_M/L > 0), and whose levels revert back closer to non-infected control levels, i.e. are recovered, (log_2_H/M < 0) in response to Ago1 knockdown, and (ii) proteins that are downregulated by *Leishmania* infection (log_2_M/L < 0) and which are recovered by Ago1 knockdown (log_2_H/M > 0), where H (heavy) represents levels in *L. donovani*-infected, Ago1 siRNA-transfected cells and M (medium) represents levels in *L. donovani*-infected, scrambled siRNA-transfected cells. The abundances of 97/331 proteins were significantly affected following Ago1 knockdown of *Leishmania*-infected macrophages, compared to corresponding control *Leishmania*-infected macrophages with normal levels of Ago1. Of these 97 proteins, the levels of 71 proteins (21 upregulated, 50 downregulated) were recovered by Ago1 knockdown as shown in **Figures 4A**, **S4B**. The overall importance of these protein modulations and recoveries were ranked by combining the Manhattan scores of *Leishmania* modulation and Ago1 modulation (as described in “Materials and methods”) and listed in descending order in **Table S2**. The proteins were also classified by GO terms in three categories: biological process, cellular component, and molecular function (**Figures 4B-D**).

It was of interest to find out how many of the Ago1-dependent *Leishmania*-modulated proteins have relevance to *Leishmania* pathogenesis. For this, we searched the literature relevant to *Leishmania*-macrophage interactions, mainly using PubMed. Of the 71 *Leishmania*-modulated Ago1-dependent proteins, 20 have previously been reported in *Leishmania* infection-related studies. Of these 20 proteins, 5 proteins were upregulated by *Leishmania* (CTSG, PFKL, SEPT9, PTGS1 and ACO1) (**Table 2**) and 15 were downregulated by *Leishmania* (CTSL, EIF3A, ALDOA, NQO1, ANXA2, P4HB, HLA-A, HSP90AB1, PRDX1, PKM, HSPH1, LMNA, HSPA5, RPL13 and HSPA4) (**Table 3**).

**Table 2:** Ago1-dependent *Leishmania*-upregulated host proteins with known pathogenic relevance.

Protein names	Gene names	Uniprot ID	Avg log_2_(M/L)	Avg log_2_(H/M)	AGO1 Interaction*	Roles related to *Leishmania*	Roles related to other pathogens
**Cathepsin G**	CTSG	P08311	1.211	-1.248	U2AF2 interacts with CTSG, AGO1 and AGO2	Significantly enriched in both vaccinated and non-vaccinated mice at late chronic visceral leishmaniasis phase (59); 33-fold upregulated in murine *L. donovani* infection (60)	*T. cruzi* (61)
**ATP-dependent 6-phosphofructokinase, liver type**	PFKL	P17858	0.142	-0.334	N/A	Upregulated in BALB/c mouse macrophages upon *L. amazonensis* infection (62); Transcription was significantly enhanced by *L. donovani* infection (63)	N/A
**Septin-9**	SEPT9	Q9UHD8	0.242	-0.204	RSP17 interacts with SEPT9 and AGO1, AGO2, AGO3	Has residues phosphorylated by *Leishmania* casein kinase 1 (L-CK1.2), which is present in *Leishmania* extracellular vesicles and is essential for parasite survival (64)	Hepatitis C virus (65); Salmonella (66)
**Glyoxylate reductase/hydroxypyruvate reductase**	GRHPR	Q9UBQ7	0.174	-0.448	N/A	N/A	*T. cruzi* (67)
**Prostaglandin G/H synthase 1**	PTGS1	P23219	0.625	-0.554	N/A	Downregulated more than two-fold in response to *L. major* infection (68)	vaccinia virus (69)
**Glia maturation factor gamma**	GMFG	O60234	0.209	-0.381	N/A	N/A	*Salmonella* (70)
**Cytoplasmic aconitate hydratase**	ACO1	P21399	0.358	-0.621	N/A	Aco1 is one of the genes related to the response to *Leishmania ssp *(71).	*Mycobacterium tuberculosis* (72)
**Aconitate hydratase, mitochondrial**	ACO2	Q99798	0.158	-0.265	N/A	N/A	*Mycobacterium tuberculosis* (72)
**Phosphatidylinositol-binding clathrin assembly protein**	PICALM	Q13492	0.045	-0.065	N/A	N/A	Enterovirus A71 (73)
**Reticulon-1;Reticulon**	RTN1	Q16799	0.176	-0.254	N/A	N/A	positive-strand RNA virus (74)
**Rho GTPase-activating protein 1**	ARHGAP1	Q07960	0.132	-0.124	N/A	N/A	*Legionella* (75)
**Hematopoietic lineage cell-specific protein**	HCLS1	P14317	0.044	-0.210	N/A	N/A	human cytomegalovirus (76); *Mycobacterium tuberculosis* (77)
**Very long-chain specific acyl-CoA dehydrogenase, mitochondrial**	ACADVL	P49748	0.150	-0.147	N/A	N/A	*T. cruzi* (67); Porcine reproductive and respiratory syndrome virus infection (78)

*All interaction data are from Pathway Commons Protein-Protein Interactions (https://www.pathwaycommons.org/).

**Table 3:** Ago1-dependent *Leishmania*-downregulated host proteins with known pathogenic relevance.

Protein names	Gene names	Uniprot ID	Avg log_2_(M/L)	Avg log_2_(H/M)	AGO1 Interaction*	Roles related to *Leishmania*	Roles related to other pathogens
**Thymidine phosphorylase**	TYMP	P19971	-0.834	1.848	N/A	N/A	SARS-CoV-2 (79)
**Protein PML (TRIM19)**	PML	P29590	-0.205	0.913	N/A	N/A	viruses (80); Lassa virus and lymphocytic choriomeningitis virus (81), human foamy virus (82); HIV (83); *Listeria monocytogenes* (84, 85)
**Cathepsin L1;Cathepsin L1 heavy chain;Cathepsin L1 light chain**	CTSL	P07711	-0.559	0.903	N/A	Crucial for a Th1-type immune response during *L. major* infection, plays a role in the processing of soluble *Leishmania* antigen (SLA) (51); Treatment with cathepsin L inhibitor potentiates Th2-type immune response in *L. major*-infected BALB/c mice (52)	SARS-CoV-2 (86); *Mycobacterium tuberculosis* (87, 88); *M. avium* and (88); mycoplasmal infection (89)
**Calreticulin**	CALR	P27797	-0.212	0.181	CALR interacts with AGO1	N/A	*Mycobacterium tuberculosis* (90, 91) and *cytomegalovirus* (91, 92).
**Protein disulfide-isomerase A4**	PDIA4	P13667	-0.129	0.259	N/A	N/A	influenza A and B virus (93); SARS-CoV-2 (86)
**Eukaryotic translation initiation factor 3 subunit A**	EIF3A	Q14152	-0.101	0.305	EIF3A interacts with AGO1	Upregulated on a transcriptional level in bone marrow-derived macrophages (BMDM) upon infection with *L. donovani* amastigotes (46)	Hepatitis E virus (HEV) (94)
**Fructose-bisphosphate aldolase A;Fructose-bisphosphate aldolase**	ALDOA	P04075	-0.160	0.088	N/A	Upregulated in *L. major-*infected bone marrow-derived macrophages (68, 95); Upregulated in BALB/c mouse macrophages upon *L. amazonensis* infection (62)	Japanese encephalitis virus (96)
**40S ribosomal protein S4, X isoform**	RPS4X	P62701	-0.223	0.114	RPS4X interacts with AGO1	N/A	Influenza virus (97)
**NAD(P)H dehydrogenase [quinone] 1**	NQO1	P15559	-0.429	0.437	N/A	NRF2, which regulates NQO1, was strongly upregulated in infection with *Leishmania*. NRF2 activation promoted parasite persistence but limited TNF-a production and tissue destruction (98).	HIV-1 Rev (99); Hepatitis B virus (100)
**40S ribosomal protein S3**	RPS3	P23396	-0.156	0.089	RPS3 interacts with AGO1	N/A	*P. aeruginosa* (101); H5N1 influenza virus (102, 103)
**6-phosphogluconate dehydrogenase, decarboxylating**	PGD	P52209	-0.284	0.357	N/A	N/A	Influenza virus (104)
**40S ribosomal protein S13**	RPS13	P62277	-0.192	0.124	RPS13 interacts with AGO1	N/A	*Chlamydia* (105)
**Annexin A6;Annexin**	ANXA6	P08133	-0.324	0.218	N/A	N/A	influenza A virus (IAV) (106, 107); *Escherichia coli* (108)
**Glutamine–fructose-6-phosphate aminotransferase [isomerizing] 1**	GFPT1	Q06210	-0.144	0.719	N/A	N/A	Adenovirus 5 (109)
**Annexin A2;Annexin;Putative annexin A2-like protein**	ANXA2; ANXA2P2	P07355	-0.128	0.240	ANXA2 interacts with AGO1	Downregulated in the presence of *L. major* infection. Two S100 family members were upregulated (S100A10, S100A11), which interact with annexins A1 and A2, forming a sophisticated Ca^2+^ sensing system (56).	Cytomegalovirus (110); human papillomavirus type 16 (111); measles virus (112)
**Protein disulfide-isomerase (PDI)**	P4HB	P07237	-0.160	0.170	P4HB interacts with AGO1	Downregulated in *L. major-*infected human macrophages (56); associates with NADPH oxidase and is required for phagocytosis of *L. chagasi* promastigotes by macrophages (113)	host-pathogen interaction (114, 115); HIV-1 (116); mouse polyomavirus (117); cholera (118)
**HLA class I histocompatibility antigen, A-2 alpha chain**	HLA-A	P01892	-0.582	0.607	N/A	Downregulated in *Leishmania*-infected THP-1 (119) and mice (120)	HIV-1 (121)
**26S proteasome non-ATPase regulatory subunit 7**	PSMD7	P51665	-0.154	0.285	N/A	N/A	SARS-CoV-2 (122)
**Elongation factor 1-alpha 1;Putative elongation factor 1-alpha-like 3;Elongation factor 1-alpha**	EEF1A1; EEF1A1P5	P68104	-0.381	0.175	EEF1A1 interacts with AGO1	N/A	HIV-1 (123); Paramyxovirus Respiratory Syncytial Virus (124)
**Protein disulfide-isomerase A3**	PDIA3	P30101	-0.229	0.200	PDIA3 interacts with AGO1	N/A	viruses (125)
**Peptidyl-prolyl cis-trans isomerase B**	PPIB	P23284	-0.226	0.267	N/A	N/A	*Burkholderia pseudomallei* (126)
**Heat shock protein HSP 90-beta**	HSP90AB1	P08238	-0.315	0.280	HSP90AB1 interacts with AGO1	Upregulated at the transcriptional level in *L. donovani* promastigote-infected macrophages (46)	HIV (127, 128)
**Peroxiredoxin-1**	PRDX1	Q06830	-0.328	0.400	PRDX1 interacts with AGO1	Upregulated at the transcriptional level in *L. donovani* promastigote-infected macrophages (46)	*Mycobacterium tuberculosis* (77, 129) (129); Respiratory Syncytial virus infection (130)
**60S ribosomal protein L17**	RPL17; RPL17-C18orf32	P18621	-0.091	0.117	RPL17 Interacts with AGO1	N/A	N/A
**Long-chain-fatty-acid–CoA ligase 3**	ACSL3	O95573	-0.425	0.381	N/A	N/A	Poliovirus (131)
**Pyruvate kinase PKM;Pyruvate kinase**	PKM	P14618	-0.190	0.122	PKM interacts with AGO1	Significantly upregulated at the transcriptional level upon *L. donovani* infection in neutrophils (63)	*Salmonella typhimurium* (132)
**Heat shock protein 105 kDa**	HSPH1	Q92598	-0.239	0.387	HSPH1 interacts with AGO1	Upregulated in bone marrow-derived macrophages (BMDMs) infected by *L. donovani* amastigotes (46)	*Helicobacter pylori* (133)
**Translation initiation factor eIF-2B subunit alpha**	EIF2B1	Q14232	-0.345	0.379	N/A	N/A	Sandfly Fever Sicilian phlebovirus (SFSV) (134)
**Elongation factor 2**	EEF2	P13639	-0.122	0.211	EEF2 interacts with AGO1	N/A	HIV-1 virus (135)
**Prelamin-A/C;Lamin-A/C**	LMNA	P02545	-0.083	0.503	LMNA interacts with AGO1	Enhances Th1 differentiation and response against *L. major* (136)	vaccinia virus (136)
**40S ribosomal protein S20**	RPS20	P60866	-0.199	0.124	RPS20 interacts with AGO1	N/A	poxvirus (137)
**ATP synthase subunit O, mitochondrial**	ATP5O	P48047	-0.097	0.045	ATP5O interacts with AGO1	N/A	N/A
**Staphylococcal nuclease domain-containing protein 1**	SND1	Q7KZF4	-0.032	0.127	Reported to be a component of RISC (138)	N/A	Kaposi’s sarcoma associated herpesvirus (KSHV) (139); chlamydial lung infection (140)
**78 kDa glucose-regulated protein (heat shock 70kDa protein 5)**	HSPA5	P11021	-0.210	0.272	HSPA5 interacts with AGO1	Upregulated in macrophages at 24h infection with *L. infantum* (141)	Ebola virus (142); Coxsackievirus A9 (143).
**Nucleoside diphosphate kinase;Nucleoside diphosphate kinase B (Ndk)**	NME1-NME2; NME2; NME1	P22392	-0.165	0.140	NME1 interacts with AGO1	N/A	Epstein-Barr virus (EBV), Human Papillomavirus (HPV) and Kaposi’s Sarcoma-associated Herpesvirus (KSHV) (144)
**Ribonuclease inhibitor**	RNH1	P13489	-0.234	0.160	N/A	N/A	SARS-CoV-2 (145); HIV-1 (146)
**60S ribosomal protein L13**	RPL13	P26373	-0.164	0.175	RPL13 interacts with AGO1	Among the top 10 downregulated genes in macrophages infected with *L. major* (147)	Foot-and-Mouth Disease virus (FMDV) (148, 149).
**60S ribosomal protein L5**	RPL5	P46777	-0.108	0.221	RPL5 interacts with AGO1	N/A	Potato Spindle Tuber Viroid (PSTVd) (150, 151)
**60S ribosomal protein L10a**	RPL10A	P62906	-0.163	0.163	RPL10A interacts with AGO1	N/A	Hepatitis C virus (HCV) and cricket paralysis virus (CrPV) (152)
**T-complex protein 1 subunit theta**	CCT8	P50990	-0.071	0.095	CCT8 interacts with AGO1	N/A	*Legionella* (75); tobamovirus infection in plants (153)
**Heat shock 70 kDa protein 4 (HSP70)**	HSPA4	P34932	-0.084	0.200	HSPA4 Interacts with AGO1	Hypoxia modulates expression of HSPA4 and reduces *Leishmania* infection in macrophages (154)	enterovirus 71 (155); dengue virus (156); Influenza A virus (157)
**Elongation factor 1-gamma**	EEF1G	P26641	-0.172	0.153	EEF1G interacts with AGO1	N/A	Foot and mouth disease virus (FMDV) (158); influenza A virus (159); HIV-1 (160)
**60S ribosomal protein L6**	RPL6	Q02878	-0.062	0.150	RPL6 interacts with AGO1	N/A	viral acute respiratory infection (ARI) in infants (161); influenza A virus (162).

*All interaction data are from Pathway Commons Protein-Protein Interactions (https://www.pathwaycommons.org/).

Together, the quantitative proteomics analysis of *Leishmania*-modulated proteins in Ago1-deficient host cells reveals that a significant number of Ago1-dependent proteins have potential implications in *Leishmania* pathogenesis based on previously published studies. How many of the remaining *Leishmania*-modulated Ago1-dependent host proteins have a role to play in *Leishmania*-infection related activities remains to be investigated.

## 4 Discussion

It is well established that *Leishmania* parasites have evolved to acquire the ability to alter the cell biology of their host cells to favor their survival and pathogenesis. Since sncRNAs are involved in post-transcriptional regulation of gene expression via RNAi, these biomolecules can play critical roles in regulating host-pathogen interactions. In this context, several recent studies have clearly shown that sncRNAs play a crucial role in microbial infections (12–14, 23) including protozoan parasite infections such as *Leishmania* infection (163–165). There is growing evidence that sncRNAs are involved in the outcome of infections. Dysregulation of host miRNA has been associated with attenuated immune response and thus pathogen survival. On the other hand, sncRNAs can be employed by the host to counter parasite survival. Extracellular vesicles seem to facilitate this communication (164).

The current study was initiated based on our hypothesis that *Leishmania* regulates the host cell RNAi process to promote its survival in infected host cells. Since Argonautes are central components of the RNAi pathway, we began by investigating whether *Leishmania* regulates the two most abundant host cell Argonautes—Ago1 and Ago2—to its advantage. We found that *Leishmania* infection selectively enhanced macrophage Ago1 expression. Strikingly, enhanced level of Ago1 in infected cells correlated with higher levels of Ago1-containing complexes, suggesting preference for Ago1 to regulate RNAi-mediated host genes regulation. Our finding of upregulated expression of Ago1 and enrichment of Ago1 complexes in *Leishmania*-infected host cells prompted us to assess the potential involvement of Ago1 in *Leishmania* survival. For this study, we knocked down Ago1 using specific siRNAs followed by parasite rescue assay. Interestingly, downregulation of host macrophage Ago1 resulted in significant reduction of *Leishmania* survival in infected cells. Together, these results strongly demonstrate the involvement of Ago1 in promoting *Leishmania* survival in infected cells. At present, we do not know the mechanism of *Leishmania-*mediated upregulation of Ago1 in infected cells. This upregulation can occur at any point in the transcription-translation process. This includes an increase in the rate of transcription, stability of mRNA, rate of translation and stability of protein.

Upregulation of Ago1 during intracellular infection is not unique to *Leishmania* infection, and changes in host RISC composition have also been reported in other infections. For example, increased expressions of Ago1 and Dicer1 were detected upon slow bee paralysis virus (SBPV) infection in bumblebees and linked to 17 differentially expressed miRNAs upon infection (166). Also, depletion of Ago1 and Dicer1 in the mosquito *Anopheles gambiae* during *Plasmodium berghei* infection led to a two-fold increase in the number of oocysts (167). In another study, Ago1 and/or 3 were required for optimal resistance to flu virus (168).

It has recently been shown in Epstein-Barr virus (EBV)-infected mammalian cells that sncRNAs other than miRNAs were selectively loaded onto Ago1, but not Ago2 (169). Indeed, differences in the affinity of sncRNAs for Argonaute members have been observed in both lower organisms and mammals. In *Drosophila*, it has been shown that perfectly matched sncRNAs duplexes are loaded onto Ago2, whereas non-perfectly matched sncRNAs sorted to Ago1 (170, 171). Similarly, in the RNAi pathway of *Caenorhabditis elegans*, sncRNA duplexes that perfectly match their target mRNA are preferentially incorporated into Argonaute RNAi defective-1 (RDE-1) and non-perfectly matched sncRNA duplexes are loaded onto Argonaute family members ALG-1 or ALG-2 (172, 173). In contrast, sncRNA-mediated selectivity of Ago proteins in mammals is not well understood. However, there are reports suggesting the existence of Ago selectivity for certain distinct sncRNAs. For example, RNA-sequencing of Ago1-, Ago2- and Ago3-associated miRNAs revealed that some have a bias toward particular Ago proteins (20). Together, these findings strongly suggest the existence of selective Ago sorting mechanisms that direct distinct sncRNAs onto specific Ago-containing RISCs, and that Ago1 may be a preferred Ago for the loading of non-perfectly matching sncRNAs, including sncRNAs from pathogens.

To further investigate the role of Ago1 in *Leishmania* infection, we used SILAC-based quantitative LC-MS/MS proteomics analysis of *L. donovani*-infected Ago1-deficient cells, which revealed differentially expressed proteins in response to *Leishmania* infection that are dependent on Ago1. As expected, many of the modulated proteins that we identified have been previously shown to be implicated in *Leishmania* infection-related processes. For example, strongly modulated proteins Ferritin heavy chain (FTH1) and Cystatin A (CSTA) were upregulated and downregulated respectively, in agreement with previous studies (40, 41, 174). Apoptosis-associated speck-like protein containing a CARD (PYCARD, also known as ASC), which was downregulated upon *Leishmania* infection in our study, has previously been reported in multiple studies covering a range of *Leishmania* species and host cell types to be differentially expressed upon *Leishmania* infection (42–44).

From the validated set of 331 *Leishmania*-modulated proteins, a subset of 71 Ago1-dependent proteins was identified. Of these, 20 were found in previous studies relevant to leishmaniasis. Of interest, among the *Leishmania*-upregulated Ago1-dependent proteins, Cathepsin G (CTSG), has been shown to be significantly enriched at the late chronic phase of visceral leishmaniasis in mice (59). Another study reported a remarkable upregulation of CTSG in murine infection with *L. donovani* (60). Among the proteins downregulated by infection and recovered by Ago1-knockdown, Cathepsin L (CTSL) has been shown to play a crucial role for a Th1-type immune response during *L. major* infection by processing of soluble *Leishmania* antigen (SLA) for presentation on MHC class II molecules (51). Further, treatment of *L. major*-infected mice with CLIK148, a specific inhibitor of CTSL, exacerbated the disease by enhancing an SLA-specific Th2-type response (51).

In addition, it was of interest to analyze the *Leishmania*-modulated Ago1-dependent proteins in relation to other intracellular pathogen infections. Interestingly, 53 out of the 71 proteins have been shown to be related to the pathogenesis of other intracellular pathogens (**Tables 2**, **3**). For example, host glyoxylate reductase/hydroxypyruvate reductase and mitochondrial very long-chain specific acyl-CoA dehydrogenase are both suppressed by the protozoan pathogen *Trypanosoma cruzi* (67). Protein promyelocytic leukemia protein/tripeptide motif protein (PML/TRIM19) has been reported to show activity against several viruses (80). Lassa virus and lymphocytic choriomeningitis virus replicate to higher levels in PML-deficient cells (81), whereas human foamy virus replication efficiency was found to be decreased upon overexpression of PML (82). Another protein of interest is calreticulin, which is known to be released onto the cell surface as an eat-me signal as a result of infection, such as in *Mycobacterium tuberculosis* and *cytomegalovirus* infections (90, 92). Furthermore, deliberate downregulation of calreticulin significantly increased the intracellular survival of *M. tuberculosis* (92).

Interestingly, based on the Pathways Commons collection of databases, 31 out of 71 *Leishmania*-modulated Ago1-knockdown-recovered proteins have reported interaction with Ago1, providing additional confidence in our Ago1-dependent proteins. We also note that 346 out of 417 proteins that were differentially expressed upon Ago1-knockdown were not modulated upon *Leishmania*-infection. Nevertheless, the data provided can act as a valuable resource for data mining by other investigators interested in Ago1 functions and its role in the RNAi pathway (**Figure S5** and **Table S1**). We note that our data is restricted to proteomic analysis. It will be of interest to complement these findings with relevant biological assays and may be considered for future studies.

**Figure 5:**
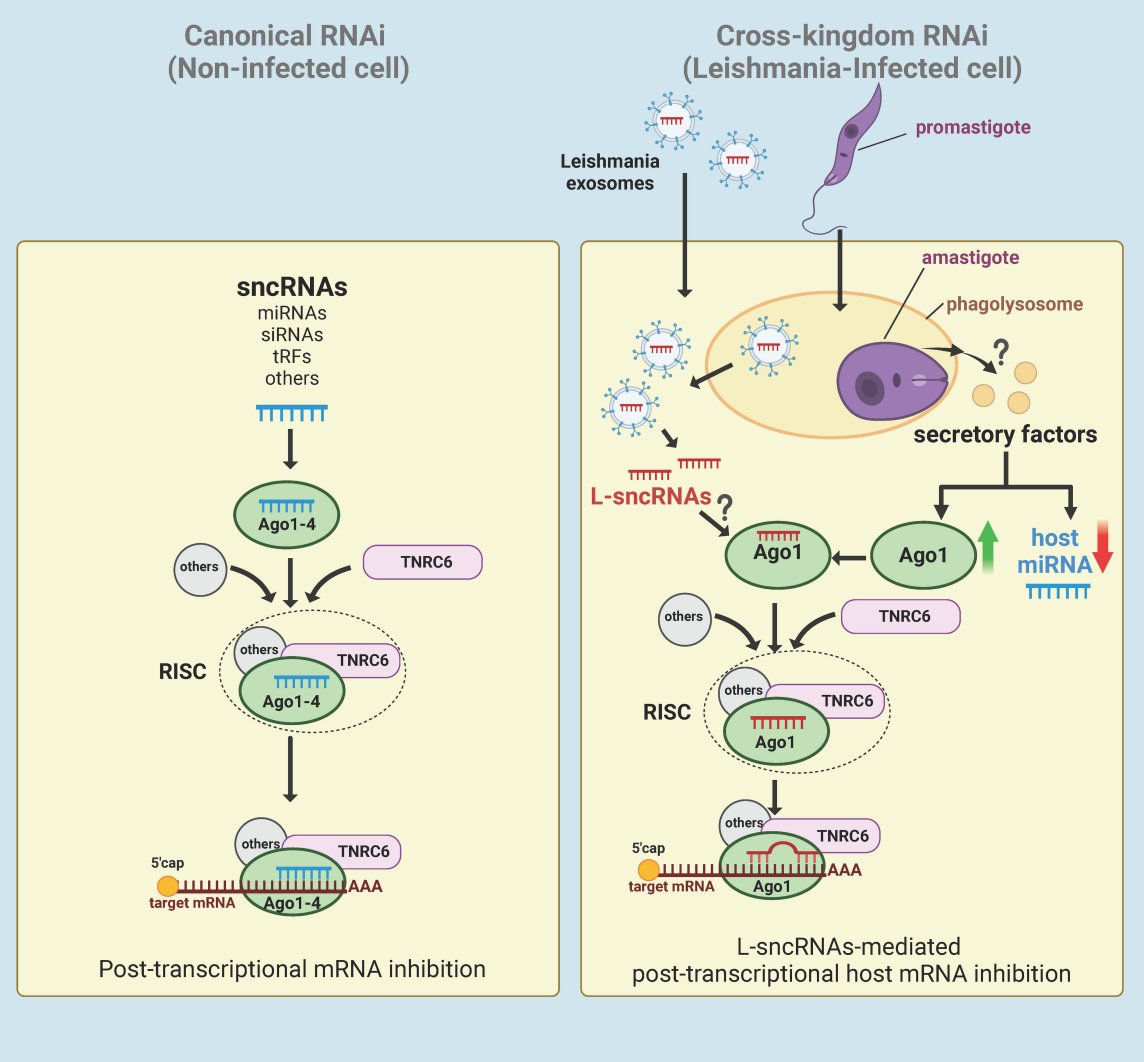
Hypothetical model of *Leishmania*-macrophage cross-kingdom RNAi. In canonical RNAi (left panel), endogenous sncRNAs interact with Ago1-4 proteins and then assembles with TNRC6 and other proteins to form the RISC. The RISC then regulates gene expression of the cell at post-transcriptional level by targeting the 3’UTR of mRNAs. In *Leishmania*-infected cells (right panel), *Leishmania* induces broad-based downregulation of host miRNAs and upregulation of Ago1 via an unknown mechanism, possibly by secretory factors. *Leishmania* exosomes containing ncRNAs secreted by amastigotes in the phagolysosomes of either the host cell or infected bystander cells are delivered to the cytosol. For RNAi, the delivered *Leishmania* exosomal sncRNAs (L-sncRNAs) are preferentially loaded onto host Ago1, which does not require full complementarity to target the mRNA. Created with BioRender.com.

Given that Ago1 knockdown results in broad effects on the host cell proteomic profile, our results generate an attractive hypothesis that *Leishmania*-mediated upregulation of Ago1 is a strategy to regulate host cell gene expression in its favour. Since Ago1 interacts directly with sncRNAs, it is probable that *Leishmania* skews host RNAi by selectively uploading sncRNAs onto Ago1, including its own sncRNAs (**Figure 5**). This model also has support of the following published findings: [1] *Leishmania* exosomes delivered to the host cytosol contain *Leishmania* sncRNAs (25); [2] *Leishmania* infection results in a broad-based downregulation of host macrophage miRNA expression, thus freeing host Agos for exogenous sncRNA loading (24); [3] Ago1 does not require full complementarity of sncRNA to target mRNA, making it favorable for the loading of pathogen sncRNAs (170, 171); [4] Unloaded Ago proteins are not stable and are degraded by the proteasome degradation pathway (175, 176) and thus, increased Ago1 abundance is presumably complemented with increased loading of sncRNAs onto Ago1 for its increased stability (176). Together, this model adds to increasing evidence of cross-kingdom RNAi in which sncRNAs are transported bi-directionally between the host and their pathogens as a host defense mechanism or strategy of pathogens to regulate host cell biology in their favor (177).

To our knowledge, this is the first report showing the role of host macrophage Ago1 in the pathogenesis of an intracellular parasitic infection. The prior understanding of the role of RNA interference mechanisms in infection is mainly based on plants and insects. These findings represent a framework for further study of the role of RNAi in *Leishmania* pathogenesis in humans. A deep understanding of cross-kingdom RNAi in context of *Leishmania* infection could provide novel therapeutic strategies for managing and treating leishmaniasis and may have implications for other intracellular pathogens.

## Data availability statement

The datasets presented in this study can be found in online repositories/**Supplementary Material**. The names of the repository/repositories and accession number(s) can be found in the article.

## Author contributions

AM: Formal Analysis, Investigation, Methodology, Validation, Writing – original draft, Writing – review & editing. SC: Data curation, Formal Analysis, Methodology, Writing – original draft, Writing – review & editing. SK: Investigation, Writing – review & editing. K-MM: Formal Analysis, Methodology, Software, Writing – review & editing. LF: Formal Analysis, Funding acquisition, Writing – review & editing. NR: Conceptualization, Funding acquisition, Project administration, Supervision, Writing – review & editing. DN: Conceptualization, Investigation, Project administration, Supervision, Validation, Writing – review & editing.

## Supplementary material

Supplementary Figure 1 Macrophage Ago1 abundance is not affected in response to phagocytosis of latex beads. dTHP-1 cells were treated with latex beads at different multiplicities of infection (MOIs) or not for 24 h, whole cell lysates from non-treated and latex beads treated macrophages were analysed by Western blotting with antibodies specific for Ago1 and Actin (loading control). Densitometric analysis was used to measure Ago1 normalized to Actin. The histogram shown is the mean ± S.D. of the densitometric analyses of three independent experiments.

Supplementary Figure 2 Preparation of recombinant proteins for GST-T6B affinity mediated pull-down assay for the isolation of Ago complexes. Recombinant GST-T6B peptide and GST alone were prepared as described in “Materials and methods”. **(A)** Purity assessment of recombinant proteins (stained with Coomassie Blue) used for the preparation of affinity beads to perform GST-T6B peptide mediated pull-down assay as described in “Materials and methods”. **(B)** The samples from purified and concentrated recombinant proteins were analyzed by Western blotting using anti-GST antibodies. **(C)** GST-T6B affinity based pull-down assay was performed to isolate Ago complexes from dTHP-1 whole cell lysates. The presence of Ago proteins was tested using indicated antibodies in a Western blot assay. GST alone affinity beads were used as a negative control for non-specific protein interaction. The result shown represents one of three independent experiments.

Supplementary Figure 3 SiRNA-mediated Ago1 knockdown in macrophages for SILAC-based comparative proteomics analysis. **(A)** Schematic diagram of metabolic SILAC quantitative proteomic strategy. **(B)** Labeled THP-1 cells were treated with an equal mix of two human Ago1 siRNAs or with control scrambled siRNA. Control and Ago1-knockdown cells were differentiated and incubated with *Leishmania* for 24 h. Subsequently, control and infected cells were washed, and whole cell lysates were prepared for SILAC-based proteomic analysis. In parallel, samples were assessed by Western blotting to confirm the downregulation of Ago1 protein levels by Ago1 siRNAs and the preservation of *Leishmania*-mediated Ago1 induction in the presence of scrambled siRNA. The fold changes are normalized to scrambled siRNA-transfected non-infected cells. Shown is the mean ± S.D. of the densitometric analyses of three independent experiments. (**p-value < 0.01).

Supplementary Figure 4 Schematic diagram detailing the data processing steps involved in the determination of **(A)** *Leishmania* infection-modulated host proteins; and **(B)** Ago1-dependent (i.e. recovered by Ago1 knockdown) Ld-modulated proteins.

Supplementary Figure 5 Quantitative proteomic analysis of Ago1 knockdown-affected host macrophage proteins in the presence of infection. **(A)** Pie chart of the log_2_(fold change) distribution of Ago1 knockdown-affected proteins. H/M normalized ratios were log_2_ transformed and averaged for at least 2 replicates. P-values < 0.05 from one-sample T-tests were considered significant. Proteins with a negative log_2_(fold change) were downregulated in *Leishmania*-infected cells treated with Ago1 siRNA compared to *Leishmania*-infected cells transfected with scrambled siRNA, whereas proteins with a positive log_2_(fold change) were upregulated in *Leishmania*-infected cells treated with Ago1 siRNA compared to *Leishmania*-infected cells treated with scrambled siRNA. **(B)** Volcano plot comparing protein expression levels in Ago1-knockdown cells with those in cells transfected with control scrambled siRNA, both infected with *Leishmania*. Proteins significantly upregulated following Ago1 knockdown are shown as green dots, and those that were downregulated, as red dots. The top 30 modulated proteins based on Manhattan distance are labeled. The horizontal dashed line marks the p-value cut off of 0.05 or -log_10_(p-value) = 1.301. The vertical dashed line represents log_2_(H/M) = 0. M: Medium (Scrambled siRNA-treated, Ld-infected), H: Heavy (Ago siRNA-treated, Ld-infected).
