# Supplementary figures and images for: Leishmania infection upregulates and engages host macrophage Argonaute 1, and system-wide proteomics reveals Argonaute 1-dependent host response
Source: Front Immunol. 2023 Nov 30;14:1287539. doi: 10.3389/fimmu.2023.1287539 (PMC10720368; doi:10.3389/fimmu.2023.1287539)

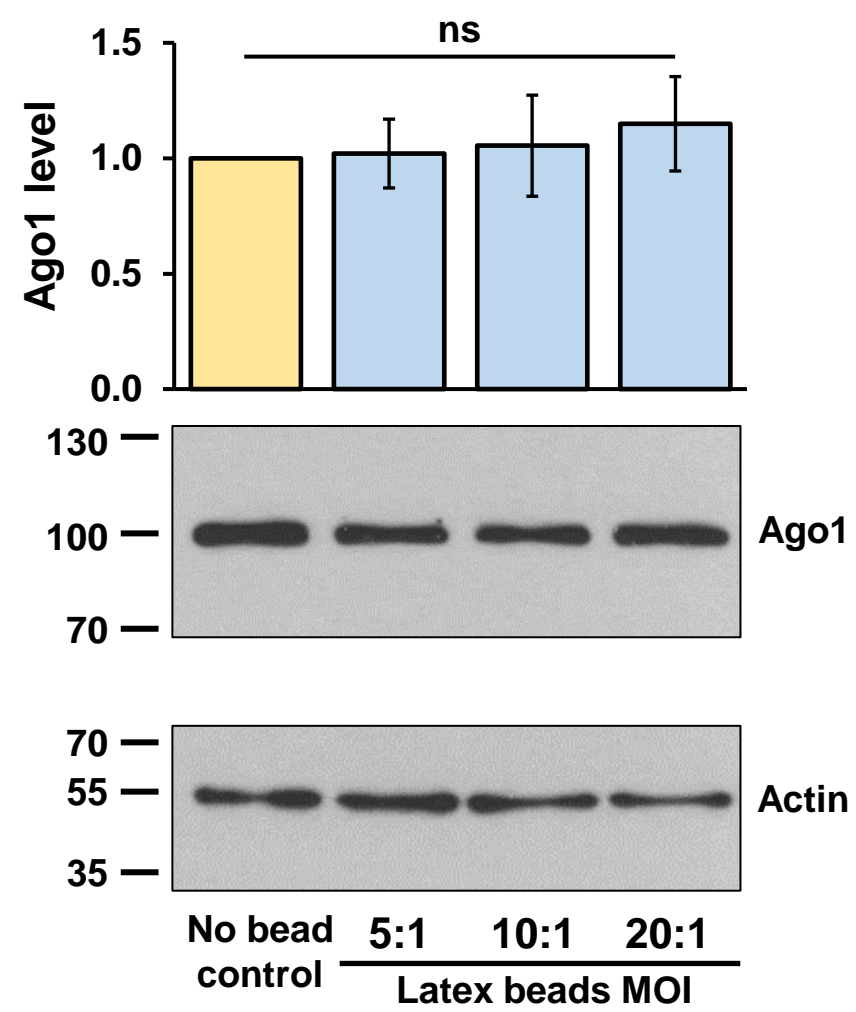

Supplement: Supplementary Figure 1 — Macrophage Ago1 abundance is not affected in response to phagocytosis of latex beads. dTHP-1 cells were treated with latex beads at different multiplicities of infection (MOIs) or not for 24 h, whole cell lysates from non-treated and latex beads treated macrophages were analysed by Western blotting with antibodies specific for Ago1 and Actin (loading control). Densitometric analysis was used to measure Ago1 normalized to Actin. The histogram shown is the mean ± S.D. of the densitometric analyses of three independent experiments. [file Image_1.pdf]

**A**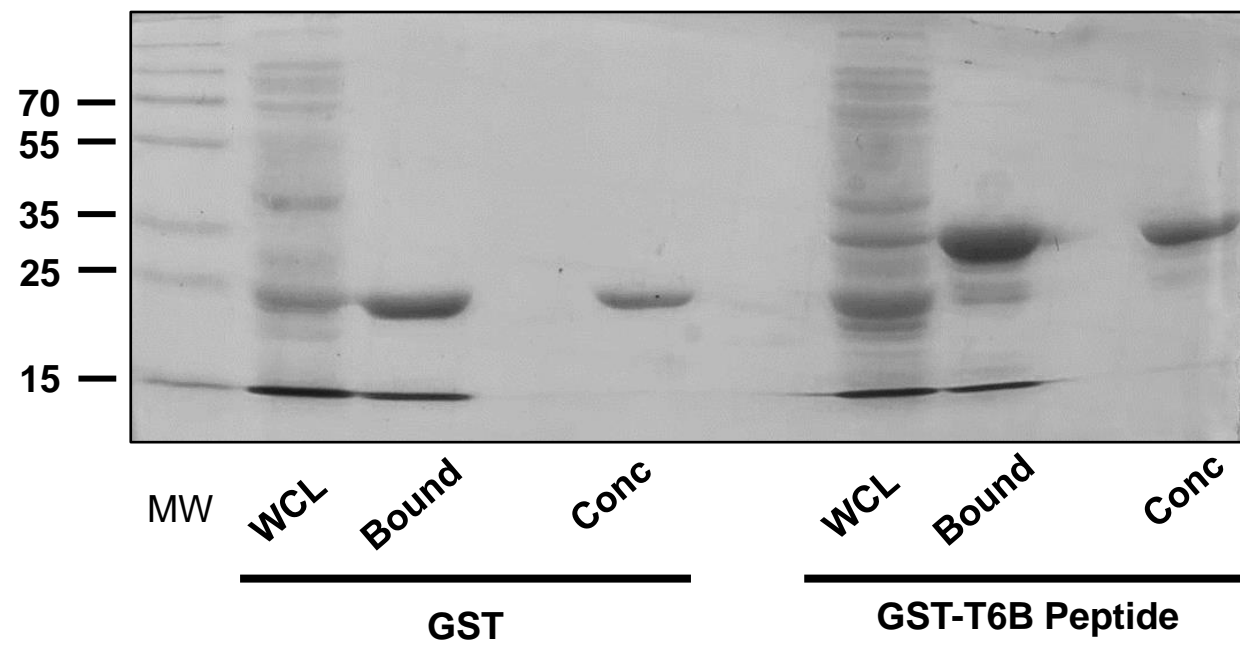**B**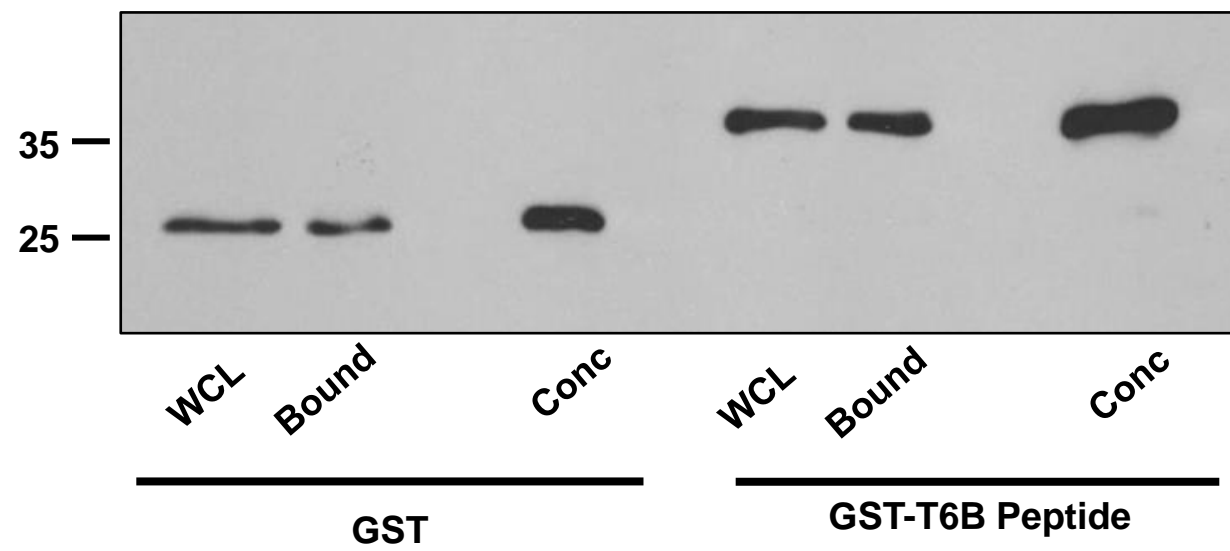**C**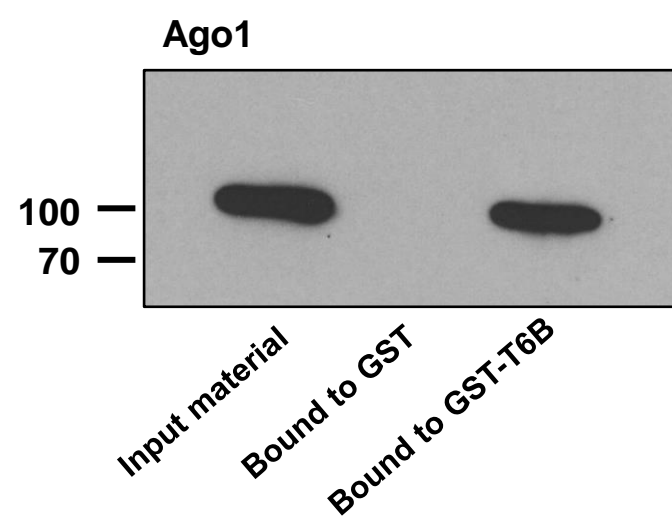**D**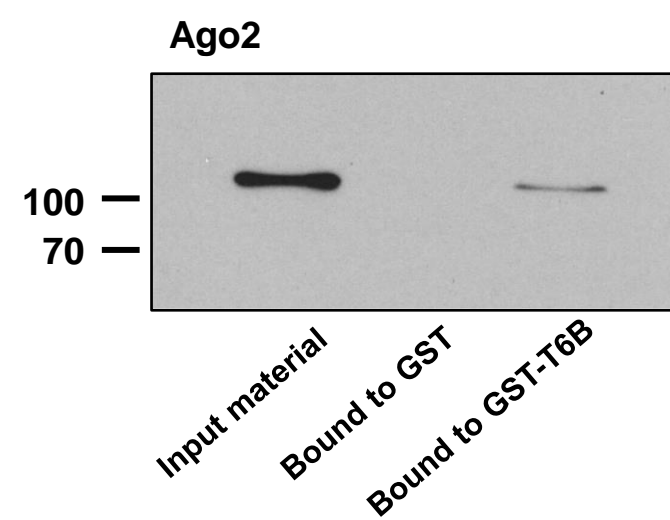

Supplement: Supplementary Figure 2 — Preparation of recombinant proteins for GST-T6B affinity mediated pull-down assay for the isolation of Ago complexes. Recombinant GST-T6B peptide and GST alone were prepared as described in “Materials and methods”. (A) Purity assessment of recombinant proteins (stained with Coomassie Blue) used for the preparation of affinity beads to perform GST-T6B peptide mediated pull-down assay as described in “Materials and methods”. (B) The samples from purified and concentrated recombinant proteins were analyzed by Western blotting using anti-GST antibodies. (C) GST-T6B affinity based pull-down assay was performed to isolate Ago complexes from dTHP-1 whole cell lysates. The presence of Ago proteins was tested using indicated antibodies in a Western blot assay. GST alone affinity beads were used as a negative control for non-specific protein interaction. The result shown represents one of three independent experiments. [file Image_2.pdf]

A

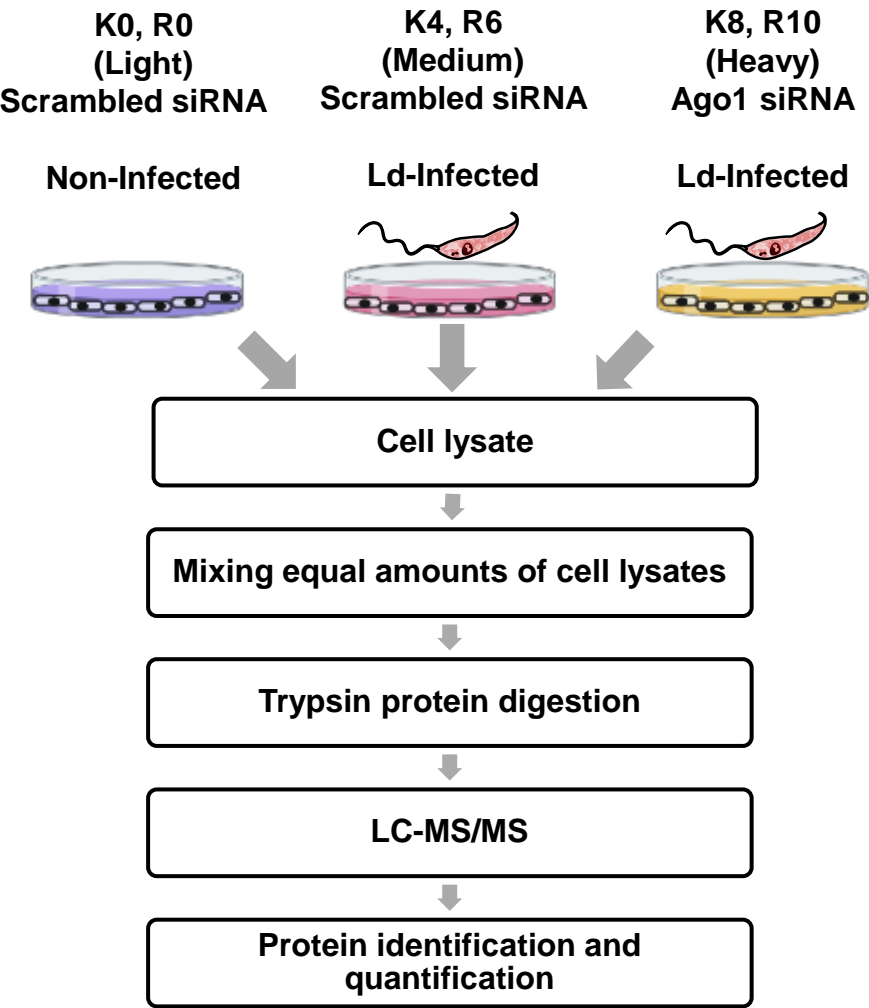

B

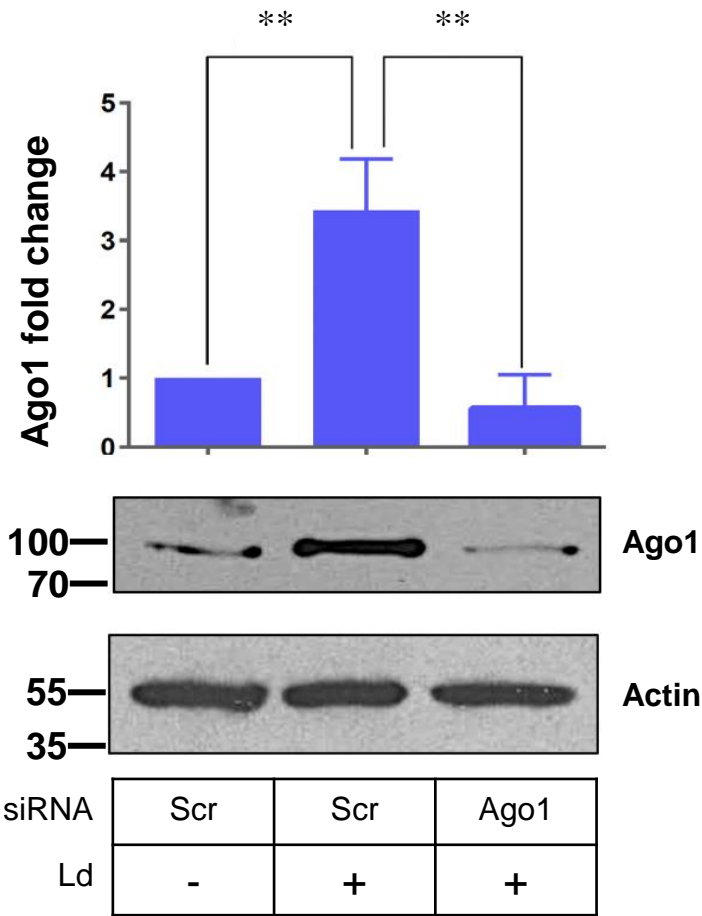

Supplement: Supplementary Figure 3 — SiRNA-mediated Ago1 knockdown in macrophages for SILAC-based comparative proteomics analysis. (A) Schematic diagram of metabolic SILAC quantitative proteomic strategy. (B) Labeled THP-1 cells were treated with an equal mix of two human Ago1 siRNAs or with control scrambled siRNA. Control and Ago1-knockdown cells were differentiated and incubated with Leishmania for 24 h. Subsequently, control and infected cells were washed, and whole cell lysates were prepared for SILAC-based proteomic analysis. In parallel, samples were assessed by Western blotting to confirm the downregulation of Ago1 protein levels by Ago1 siRNAs and the preservation of Leishmania-mediated Ago1 induction in the presence of scrambled siRNA. The fold changes are normalized to scrambled siRNA-transfected non-infected cells. Shown is the mean ± S.D. of the densitometric analyses of three independent experiments. (**p-value < 0.01). [file Image_3.pdf]

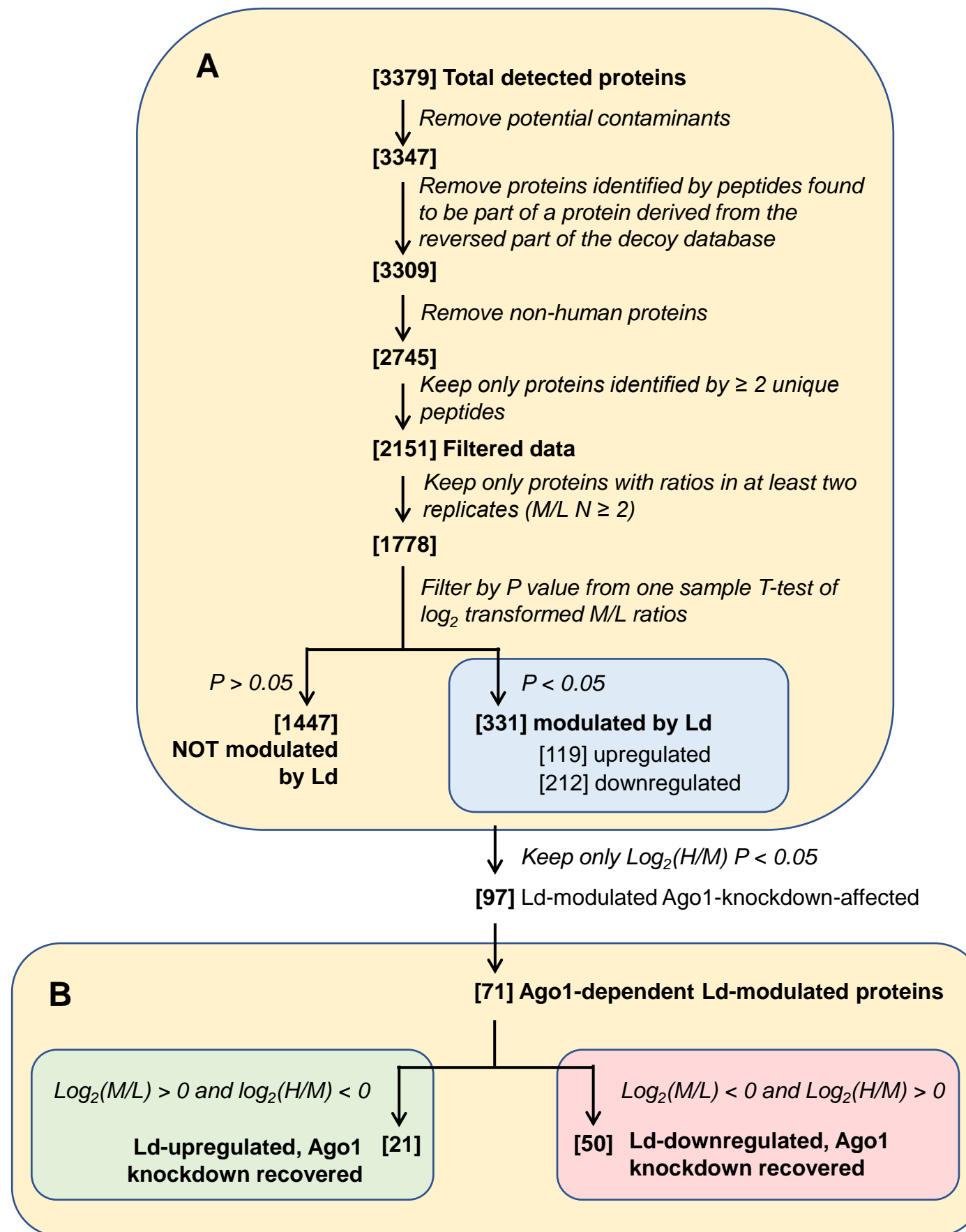

Supplement: Supplementary Figure 4 — Schematic diagram detailing the data processing steps involved in the determination of (A) Leishmania infection-modulated host proteins; and (B) Ago1-dependent (i.e. recovered by Ago1 knockdown) Ld-modulated proteins. [file Image_4.pdf]

**A**

Ago1 knockdown-affected proteins (N = 417)

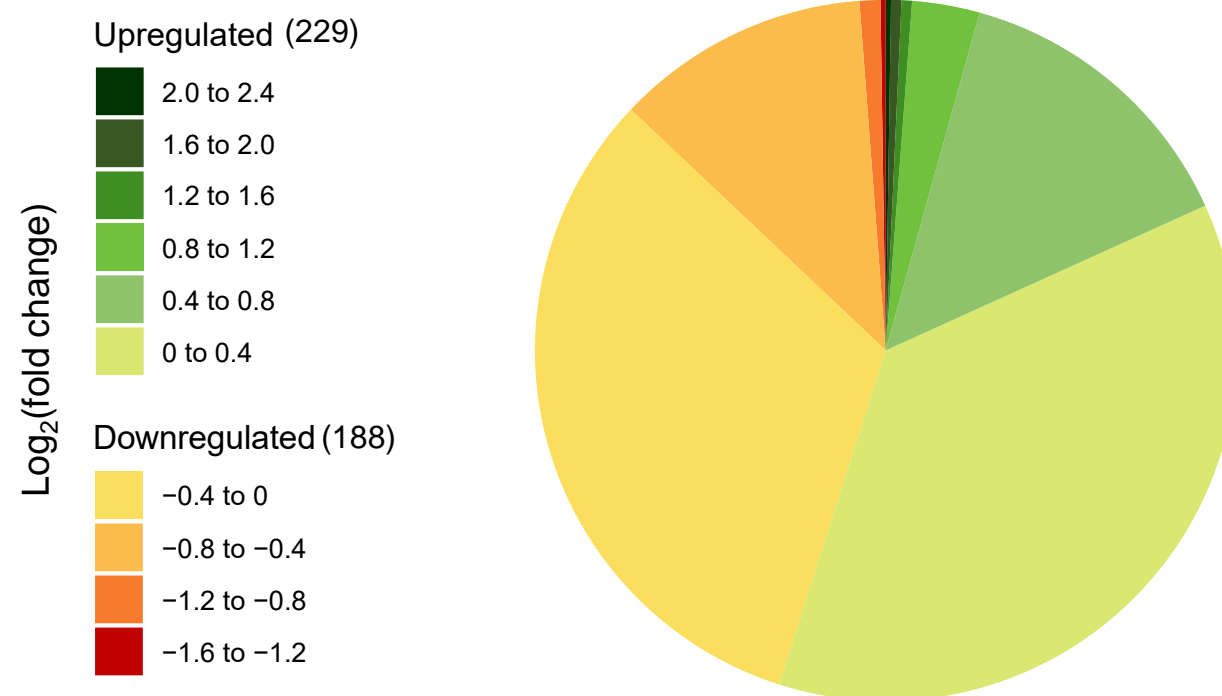**B**

Ago1 knockdown-affected proteins (N = 417)

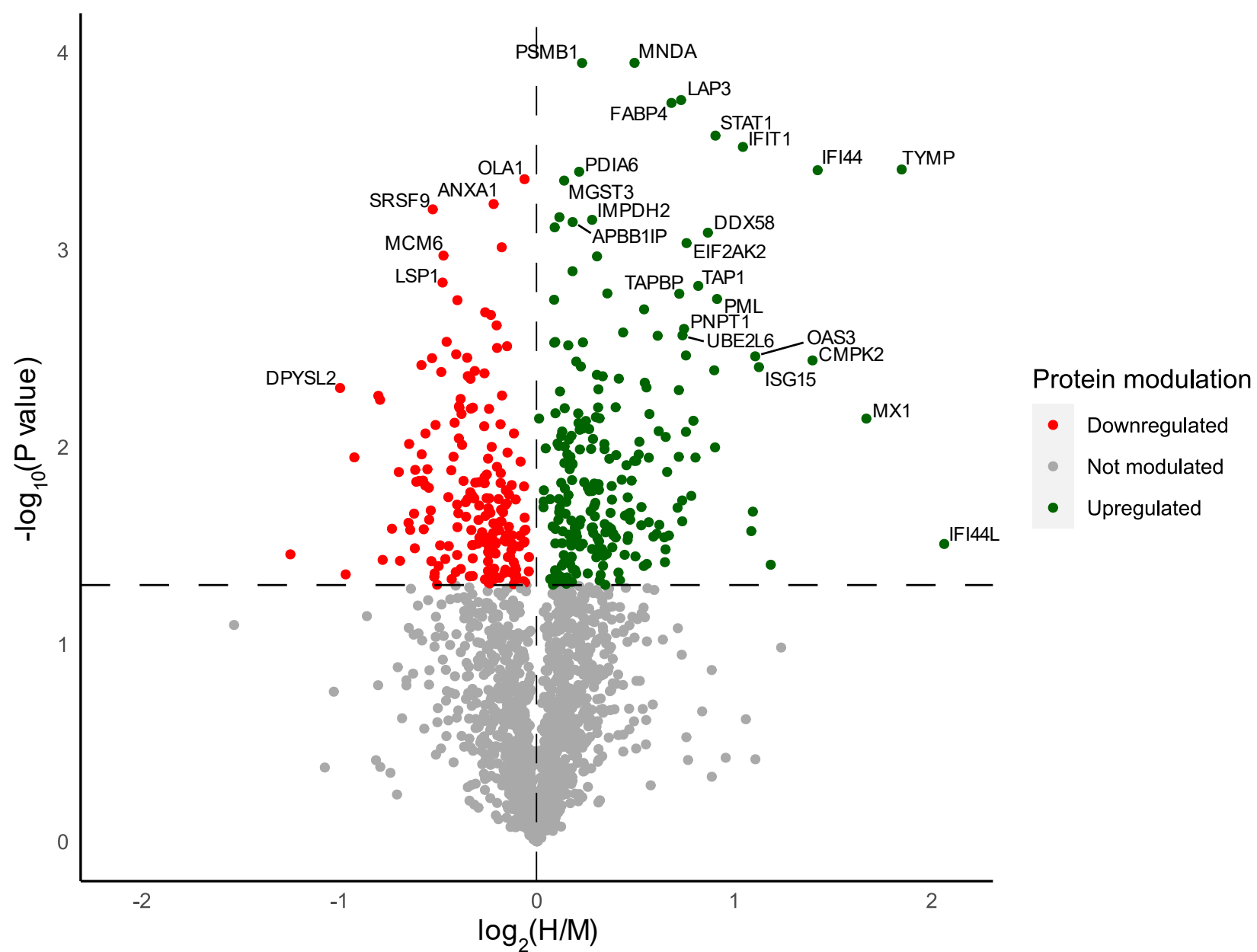

Supplement: Supplementary Figure 5 — Quantitative proteomic analysis of Ago1 knockdown-affected host macrophage proteins in the presence of infection. (A) Pie chart of the log2(fold change) distribution of Ago1 knockdown-affected proteins. H/M normalized ratios were log2 transformed and averaged for at least 2 replicates. P-values < 0.05 from one-sample T-tests were considered significant. Proteins with a negative log2(fold change) were downregulated in Leishmania-infected cells treated with Ago1 siRNA compared to Leishmania-infected cells transfected with scrambled siRNA, whereas proteins with a positive log2(fold change) were upregulated in Leishmania-infected cells treated with Ago1 siRNA compared to Leishmania-infected cells treated with scrambled siRNA. (B) Volcano plot comparing protein expression levels in Ago1-knockdown cells with those in cells transfected with control scrambled siRNA, both infected with Leishmania. Proteins significantly upregulated following Ago1 knockdown are shown as green dots, and those that were downregulated, as red dots. The top 30 modulated proteins based on Manhattan distance are labeled. The horizontal dashed line marks the p-value cut off of 0.05 or -log10(p-value) = 1.301. The vertical dashed line represents log2(H/M) = 0. M: Medium (Scrambled siRNA-treated, Ld-infected), H: Heavy (Ago siRNA-treated, Ld-infected). [file Image_5.pdf]
