# Supplementary material for: Leishmania infection upregulates and engages host macrophage Argonaute 1, and system-wide proteomics reveals Argonaute 1-dependent host response
Source: Front Immunol. 2023 Nov 30;14:1287539. doi: 10.3389/fimmu.2023.1287539 (PMC10720368; doi:10.3389/fimmu.2023.1287539)
Supplement: Supplementary file 7 [file Table_2.pdf]

Table S2. Ago1-dependent *Leishmania*-modulated host proteins

Protein names	Gene names	Uniprot ID	Avg log_2(M/L)	P-value log_2(M/L)	Avg log_2(H/M)	P-value log_2(H/M)	M/L + H/M Manhattan distance	Function*
L. donovani **infection-upregulated Ago1-knockdown affected**								
**cAMP-dependent protein kinase type II-alpha regulatory subunit**	PRKAR2A	P13861	0.373	0.008	-0.531	0.004	5.449	Regulatory subunit of the cAMP-dependent protein kinases involved in cAMP signaling. Type II regulatory chains mediate membrane association by binding to anchoring proteins, including the MAP2 kinase.
**Dihydropyrimidinase-related protein 2**	DPYSL2	Q16555	0.612	0.039	-0.996	0.005	5.317	Plays a role in neuronal development and polarity, as well as in axon growth and guidance, neuronal growth cone collapse and cell migration. May play a role in endocytosis.
**Cathepsin G**	CTSG	P08311	1.211	0.048	-1.248	0.035	5.238	Serine protease with trypsin- and chymotrypsin-like specificity
**ATP-dependent 6-phosphofructokinase, liver type**	PFKL	P17858	0.142	0.004	-0.334	0.006	5.064	Catalyzes the phosphorylation of D-fructose 6-phosphate to fructose 1,6-bisphosphate by ATP, the first committing step of glycolysis. Negatively regulates the phagocyte oxidative burst in response to bacterial infection.
**Septin-9**	SEPT9	Q9UHD8	0.242	0.034	-0.204	0.002	4.536	Filament-forming cytoskeletal GTPase (By similarity). May play a role in cytokinesis. May play a role in the internalization of 2 intracellular microbial pathogens, *Listeria monocytogenes* and *Shigella flexneri*
**Glyoxylate reductase/ hydroxypyruvate reductase**	GRHPR	Q9UBQ7	0.174	0.007	-0.448	0.018	4.494	Enzyme with hydroxy-pyruvate reductase, glyoxylate reductase and D-glycerate dehydrogenase enzymatic activities.
**Prostaglandin G/H synthase 1**	PTGS1	P23219	0.625	0.049	-0.554	0.013	4.373	Dual cyclooxygenase and peroxidase in the biosynthesis pathway of prostanoids, with a particular role in the inflammatory response.
**Glia maturation factor gamma**	GMFG	O60234	0.209	0.028	-0.381	0.007	4.305	N/A
**Exosome complex component RRP41**	EXOSC4	Q9NPD3	0.297	0.043	-0.699	0.013	4.241	Non-catalytic component of the RNA exosome complex which has 3'->5' exoribonuclease activity and participates in a multitude of cellular RNA processing and degradation events.
**Cytoplasmic aconitate hydratase**	ACO1	P21399	0.358	0.028	-0.621	0.022	4.188	Bifunctional iron sensor that switches between 2 activities depending on iron availability.
**Gamma-glutamylcyclotransferase**	GGCT	O75223	0.206	0.037	-0.616	0.013	4.138	Catalyzes the formation of 5-oxoproline from gamma-glutamyl dipeptides and may play a significant role in glutathione homeostasis. Induces release of cytochrome c from mitochondria with resultant induction of apoptosis.
**Aconitate hydratase, mitochondrial**	ACO2	Q99798	0.158	0.022	-0.265	0.014	3.938	Catalyzes the isomerization of citrate to isocitrate via cis-aconitate.
**Phosphatidylinositol-binding clathrin assembly protein**	PICALM	Q13492	0.045	0.010	-0.065	0.016	3.890	Cytoplasmic adapter protein that plays a critical role in clathrin-mediated endocytosis which is important in processes such as internalization of cell receptors, synaptic transmission or removal of apoptotic cells.
**Golgi-associated plant pathogenesis-related protein 1**	GLIPR2	Q9H4G4	0.475	0.027	-0.431	0.047	3.801	N/A
**Histidine triad nucleotide-binding protein 2, mitochondrial**	HINT2	Q9BX68	0.212	0.017	-0.247	0.038	3.655	Exhibits adenosine 5'-monophosphoramidase activity.

Protein names	Gene names	Uniprot ID	Avg log_2(M/L)	P-value log_2(M/L)	Avg log_2(H/M)	P-value log_2(H/M)	M/L + H/M Manhattan distance	Function*
**Reticulon-1;Reticulon**	RTN1	Q16799	0.176	0.046	-0.254	0.018	3.512	Inhibits amyloid precursor protein processing, probably by blocking BACE1 activity.
**NAD(P) transhydrogenase, mitochondrial**	NNT	Q13423	0.098	0.031	-0.327	0.031	3.444	Performs transhydrogenation between NADH and NADP, which is coupled to respiration and ATP hydrolysis, and functions as a proton pump across the membrane.
**Peptidyl-prolyl cis-trans isomerase FKBP8;Peptidyl-prolyl cis-trans isomerase**	FKBP8	Q14318	0.297	0.022	-0.078	0.049	3.351	Constitutively inactive PPiase, which becomes active when bound to calmodulin and calcium. Seems to act as a chaperone for BCL2.
**Rho GTPase-activating protein 1**	ARHGAP1	Q07960	0.132	0.028	-0.124	0.031	3.311	GTPase activator for the Rho, Rac and Cdc42 proteins, converting them to the putatively inactive GDP-bound state.
**Hematopoietic lineage cell-specific protein**	HCLS1	P14317	0.044	0.040	-0.210	0.031	3.157	Substrate of the antigen receptor-coupled tyrosine kinase. Plays a role in antigen receptor signaling for both clonal expansion and deletion in lymphoid cells.
**Very long-chain specific acyl-CoA dehydrogenase, mitochondrial**	ACADVL	P49748	0.150	0.046	-0.147	0.036	3.080	One of the acyl-CoA dehydrogenases that catalyze the first step of mitochondrial fatty acid beta-oxidation, an aerobic process allowing the production of energy from fats
***L. donovani* infection-downregulated Ago1-knockdown affected**								
**Thymidine phosphorylase**	TYMP	P19971	-0.834	0.003	1.848	0.000	8.671	Catalyzes the reversible phosphorolysis of thymidine. The produced molecules are then utilized as carbon and energy sources or in the rescue of pyrimidine bases for nucleotide synthesis.
**Protein PML (TRIM19)**	PML	P29590	-0.205	0.001	0.913	0.002	7.074	Functions via its association with PML-nuclear bodies in a wide range of cellular processes, including tumor suppression, transcriptional regulation, apoptosis, senescence, DNA damage response, and viral defense mechanisms.
**Cytosol aminopeptidase**	LAP3	P28838	-0.131	0.012	0.731	0.000	6.540	Cytosolic metallopeptidase that catalyzes the removal of unsubstituted N-terminal hydrophobic amino acids from various peptides.
**Cathepsin L1;Cathepsin L1 heavy chain;Cathepsin L1 light chain**	CTSL	P07711	-0.559	0.004	0.903	0.010	5.887	Thiol protease important for the overall degradation of proteins in lysosomes (Probable). Plays a critical for normal cellular functions such as general protein turnover, antigen processing and bone remodeling.
**Serpin H1**	SERPINH1	P50454	-0.394	0.004	0.570	0.007	5.500	Binds specifically to collagen. Could be involved as a chaperone in the biosynthetic pathway of collagen.
**Protein disulfide-isomerase A6**	PDIA6	Q15084	-0.188	0.026	0.214	0.000	5.389	May function as a chaperone that inhibits aggregation of misfolded proteins; Negatively regulates the unfolded protein response (UPR) through binding to UPR sensors such as ERN1.
**Syntenin-1**	SDCBP	O00560	-0.481	0.005	0.567	0.011	5.319	Multifunctional adapter protein involved in diverse array of functions including trafficking of transmembrane proteins, neuro and immunomodulation, exosome biogenesis, and tumorigenesis
**Calreticulin**	CALR	P27797	-0.212	0.001	0.181	0.012	5.261	Calcium-binding chaperone that promotes folding, oligomeric assembly and quality control in the endoplasmic reticulum (ER) via the calreticulin/calnexin cycle.
**Protein disulfide-isomerase A4**	PDIA4	P13667	-0.129	0.001	0.259	0.016	5.171	Catalyzes the rearrangement of -S-S- bonds in proteins.

Protein names	Gene names	Uniprot ID	Avg log_2(M/L)	P-value log_2(M/L)	Avg log_2(H/M)	P-value log_2(H/M)	M/L + H/M Manhattan distance	Function*
**Eukaryotic translation initiation factor 3 subunit A**	EIF3A	Q14152	-0.101	0.016	0.305	0.001	5.159	RNA-binding component of the eukaryotic translation initiation factor 3 (eIF-3) complex, which is required for several steps in the initiation of protein synthesis.
**Fructose-bisphosphate aldolase A;Fructose-bisphosphate aldolase**	ALDOA	P04075	-0.160	0.008	0.088	0.002	5.109	Catalyzes the reversible conversion of beta-D-fructose 1,6-bisphosphate (FBP) into two triose phosphate and plays a key role in glycolysis and gluconeogenesis.
**40S ribosomal protein S4, X isoform**	RPS4X	P62701	-0.223	0.025	0.114	0.001	5.104	Component of the ribosome.
**NAD(P)H dehydrogenase [quinone] 1**	NQO1	P15559	-0.429	0.029	0.437	0.003	4.988	Flavin-containing quinone reductase that catalyzes two-electron reduction of quinones to hydroquinones using either NADH or NADPH as electron donors.
**40S ribosomal protein S3**	RPS3	P23396	-0.156	0.007	0.089	0.003	4.916	Involved in translation as a component of the 40S small ribosomal subunit.
**6-phosphogluconate dehydrogenase, decarboxylating**	PGD	P52209	-0.284	0.044	0.357	0.002	4.776	Catalyzes the oxidative decarboxylation of 6-phosphogluconate to ribulose 5-phosphate and CO2, with concomitant reduction of NADP to NADPH.
**40S ribosomal protein S13**	RPS13	P62277	-0.192	0.004	0.124	0.009	4.738	Component of the ribosome.
**Annexin A6;Annexin**	ANXA6	P08133	-0.324	0.002	0.218	0.032	4.732	May associate with CD21. May regulate the release of Ca2+ from intracellular stores.
**Glutamine--fructose-6-phosphate aminotransferase [isomerizing] 1**	GFPT1	Q06210	-0.144	0.014	0.719	0.011	4.664	Controls the flux of glucose into the hexosamine pathway. Most likely involved in regulating the availability of precursors for N- and O-linked glycosylation of proteins.
**Annexin A2;Annexin;Putative annexin A2-like protein**	ANXA2; ANXA2P2	P07355	-0.128	0.004	0.240	0.015	4.609	Calcium-regulated membrane-binding protein whose affinity for calcium is greatly enhanced by anionic phospholipids. May be involved in heat-stress response.
**Protein disulfide-isomerase**	P4HB	P07237	-0.160	0.005	0.170	0.011	4.569	This multifunctional protein catalyzes the formation, breakage and rearrangement of disulfide bonds.
**HLA class I histocompatibility antigen, A-2 alpha chain**	HLA-A	P01892	-0.582	0.018	0.607	0.028	4.496	Antigen-presenting major histocompatibility complex class I (MHCI) molecule.
**Proteasome activator complex subunit 2**	PSME2	Q9UL46	-0.247	0.005	0.445	0.035	4.411	Implicated in immunoproteasome assembly and required for efficient antigen processing. The PA28 activator complex enhances the generation of class I binding peptides by altering the cleavage pattern of the proteasome.
**26S proteasome non-ATPase regulatory subunit 7**	PSMD7	P51665	-0.154	0.007	0.285	0.016	4.381	Component of the 26S proteasome, a multiprotein complex involved in the ATP-dependent degradation of ubiquitinated proteins.
**Elongation factor 1-alpha 1;Putative elongation factor 1-alpha-like 3;Elongation factor 1-alpha**	EEF1A1; EEF1A1P5	P68104	-0.381	0.005	0.175	0.032	4.331	Promotes the GTP-dependent binding of aminoacyl-tRNA to the A-site of ribosomes during protein biosynthesis. Plays a role in the positive regulation of IFNG transcription in T-helper 1 cells as part of an IFNG promoter-binding complex with TXK and PARP1
**Protein disulfide-isomerase A3**	PDIA3	P30101	-0.229	0.034	0.200	0.004	4.327	Disulfide isomerase which catalyzes the formation, isomerization, and reduction or oxidation of disulfide bonds.
**Peptidyl-prolyl cis-trans isomerase B**	PPIB	P23284	-0.226	0.010	0.267	0.017	4.287	PPIase that catalyzes the cis-trans isomerization of proline imidic peptide bonds in oligopeptides and may therefore assist protein folding.

Protein names	Gene names	Uniprot ID	Avg $log_2(M/L)$	P-value $log_2(M/L)$	Avg $log_2(H/M)$	P-value $log_2(H/M)$	M/L + H/M Manhattan distance	Function*
**Heat shock protein HSP 90-beta**	HSP90AB1	P08238	-0.315	0.006	0.280	0.033	4.277	Molecular chaperone that promotes the maturation, structural maintenance and proper regulation of specific target proteins involved for instance in cell cycle control and signal transduction.
**Peroxiredoxin-1**	PRDX1	Q06830	-0.328	0.013	0.400	0.029	4.146	Thiol-specific peroxidase that catalyzes the reduction of hydrogen peroxide and organic hydroperoxides to water and alcohols, respectively. Plays a role in cell protection against oxidative stress by detoxifying peroxides and as sensor of hydrogen peroxide-mediated signaling events.
**Myeloid-derived growth factor**	MYDGF	Q969H8	-0.144	0.027	0.402	0.011	4.074	Bone marrow-derived monocyte and paracrine-acting protein that promotes cardiac myocyte survival and adaptive angiogenesis for cardiac protection and/or repair after myocardial infarction (MI). Stimulates endothelial cell proliferation through a MAPK1/3-, STAT3- and CCND1-mediated signaling pathway. Inhibits cardiac myocyte apoptosis in a PI3K/AKT-dependent signaling pathway (By similarity).
**60S ribosomal protein L17**	RPL17; RPL17-C18orf32	P18621	-0.091	0.028	0.117	0.005	4.052	Component of the large ribosomal subunit.
**Long-chain-fatty-acid--CoA ligase 3**	ACSL3	O95573	-0.425	0.033	0.381	0.018	4.022	Acyl-CoA synthetases (ACSL) activate long-chain fatty acids for both synthesis of cellular lipids, and degradation via beta-oxidation
**Pyruvate kinase PKM;Pyruvate kinase**	PKM	P14618	-0.190	0.005	0.122	0.045	3.974	Controls the rate of glycolysis. Catalyzes the final rate-limiting step of glycolysis by mediating the transfer of a phosphoryl group from phosphoenolpyruvate (PEP) to ADP, generating ATP
**Heat shock protein 105 kDa**	HSPH1	Q92598	-0.239	0.017	0.387	0.028	3.960	Acts as a nucleotide-exchange factor (NEF) for chaperone proteins HSPA1A and HSPA1B, promoting the release of ADP from HSPA1A/B thereby triggering client/substrate protein release
**Translation initiation factor eIF-2B subunit alpha**	EIF2B1	Q14232	-0.345	0.038	0.379	0.016	3.945	Catalyzes the exchange of eukaryotic initiation factor 2-bound GDP for GTP.
**Elongation factor 2**	EEF2	P13639	-0.122	0.040	0.211	0.008	3.857	Catalyzes the GTP-dependent ribosomal translocation step during translation elongation.
**Prelamin-A/C;Lamin-A/C**	LMNA	P02545	-0.083	0.046	0.503	0.012	3.857	Components of the nuclear lamina.
**40S ribosomal protein S20**	RPS20	P60866	-0.199	0.022	0.124	0.015	3.798	Component of the ribosome
**ATP synthase subunit O, mitochondrial**	ATP5O	P48047	-0.097	0.028	0.045	0.010	3.683	Mitochondrial membrane ATP synthase (F1F0 ATP synthase or Complex V) produces ATP from ADP in the presence of a proton gradient across the membrane which is generated by electron transport complexes of the respiratory chain.
**Staphylococcal nuclease domain-containing protein 1**	SND1	Q7KZF4	-0.032	0.008	0.127	0.040	3.645	Endonuclease that mediates miRNA decay of both protein-free and AGO2-loaded miRNAs
**78 kDa glucose-regulated protein**	HSPA5	P11021	-0.210	0.033	0.272	0.021	3.635	Endoplasmic reticulum chaperone that plays a key role in protein folding and quality control in the endoplasmic reticulum lumen
**Nucleoside diphosphate kinase;Nucleoside diphosphate kinase B**	NME1-NME2; NME2; NME1	P22392	-0.165	0.042	0.140	0.012	3.605	Major role in the synthesis of nucleoside triphosphates other than ATP. Acts as a transcriptional activator of the MYC gene
**Ribonuclease inhibitor**	RNH1	P13489	-0.234	0.037	0.160	0.017	3.583	Ribonuclease inhibitor which inhibits RNASE1, RNASE2 and ANG. May play a role in redox homeostasis.
**Aldose reductase**	AKR1B1	P15121	-0.274	0.034	0.068	0.018	3.549	Catalyzes the NADPH-dependent reduction of a wide variety of carbonyl-containing compounds to their corresponding alcohols.

Protein names	Gene names	Uniprot ID	Avg $\log_2$(M/L)	P-value $\log_2$(M/L)	Avg $\log_2$(H/M)	P-value $\log_2$(H/M)	M/L + H/M Manhattan distance	Function*
**60S ribosomal protein L13**	RPL13	P26373	-0.164	0.027	0.175	0.024	3.534	Component of the ribosome, a large ribonucleoprotein complex responsible for the synthesis of proteins in the cell
**60S ribosomal protein L5**	RPL5	P46777	-0.108	0.036	0.221	0.023	3.406	Component of the ribosome, a large ribonucleoprotein complex responsible for the synthesis of proteins in the cell.
**60S ribosomal protein L10a**	RPL10A	P62906	-0.163	0.034	0.163	0.028	3.357	Component of the large ribosomal subunit.
**T-complex protein 1 subunit theta**	CCT8	P50990	-0.071	0.022	0.095	0.043	3.183	Component of the chaperonin-containing T-complex (TRiC), a molecular chaperone complex that assists the folding of proteins upon ATP hydrolysis
**Heat shock 70 kDa protein 4**	HSPA4 (HSP70)	P34932	-0.084	0.039	0.200	0.044	3.056	Chaperone-mediated protein complex assembly
**Elongation factor 1-gamma**	EEF1G	P26641	-0.172	0.043	0.153	0.046	3.027	Probably plays a role in anchoring the complex to other cellular components.
**60S ribosomal protein L6**	RPL6	Q02878	-0.062	0.031	0.150	0.049	3.026	Component of the large ribosomal subunit.

*from Uniprot (uniprot.org)
